# Spatial vs. Temporal Features in ICA of Resting-State fMRI – A Quantitative and Qualitative Investigation in the Context of Response Inhibition

**DOI:** 10.1371/journal.pone.0066572

**Published:** 2013-06-18

**Authors:** Lixia Tian, Yazhuo Kong, Juejing Ren, Gaël Varoquaux, Yufeng Zang, Stephen M. Smith

**Affiliations:** 1 Department of Biomedical Engineering, School of Computer and Information Technology, Beijing Jiaotong University, Beijing, China; 2 FMRIB (Oxford University Centre for Functional MRI of the Brain), Nuffield Dept. Clinical Neurosciences, University of Oxford, Oxford, United Kingdom; 3 State Key Laboratory of Cognitive Neuroscience and Learning, Beijing Normal University, Beijing, China; 4 Parietal team, INRIA Saclay-Ile-de-France, Saclay, France; 5 Center for Cognition and Brain Disorders, Affiliated Hospital, Hangzhou Normal University, Hangzhou, Zhejiang, China; Wake Forest School of Medicine, United States of America

## Abstract

Independent component analysis (ICA) can identify covarying functional networks in the resting brain. Despite its relatively widespread use, the potential of the temporal information (unlike spatial information) obtained by ICA from resting state fMRI (RS-fMRI) data is not always fully utilized. In this study, we systematically investigated which features in ICA of resting-state fMRI relate to behaviour, with stop signal reaction time (SSRT) in a stop-signal task taken as a test case. We did this by correlating SSRT with the following three kinds of measure obtained from RS-fMRI data: (1) the amplitude of each resting state network (RSN) (evaluated by the standard deviation of the RSN timeseries), (2) the temporal correlation between every pair of RSN timeseries, and (3) the spatial map of each RSN. For multiple networks, we found significant correlations not only between SSRT and spatial maps, but also between SSRT and network activity amplitude. Most of these correlations are of functional interpretability. The temporal correlations between RSN pairs were of functional significance, but these correlations did not appear to be very sensitive to finding SSRT correlations. In addition, we also investigated the effects of the decomposition dimension, spatial smoothing and Z-transformation of the spatial maps, as well as the techniques for evaluating the temporal correlation between RSN timeseries. Overall, the temporal information acquired by ICA enabled us to investigate brain function from a complementary perspective to the information provided by spatial maps.

## Introduction

Functional network analysis based on resting state fMRI (RS-fMRI) is rapidly emerging as a powerful tool for in vivo mapping of neural circuitry in the human brain [1]. Among various approaches for identifying and characterising resting state networks (RSNs), seed-based correlation is the most widely used. This method identifies resting state networks by detecting voxels whose time series significantly correlate with that of a pre-defined region of interest (ROI). In addition to generating highly repeatable network patterns [2], [3], seed-based correlation analysis has also been widely used to investigate the pathologies of various diseases [4] as well as the neural basis of individual differences in behaviour [5], [6] and personality traits [7]–[9].

Though seed-based correlation has proven to be a powerful and effective tool in identifying and characterising RSNs, the networks obtained from seed-based correlation are dependent upon the way the seed regions are defined [10]. Moreover, seed-based correlation only evaluates the relationship between the brain and the seed, one seed at a time, and hence overlapping functional networks are not well modelled. Independent component analysis (ICA) can be a powerful alternative for exploring RSNs [11], [12]. ICA aims to separate independent patterns by maximizing the mutual spatial independence among components. To date, ICA has been adopted not only to characterize brain function of normal subjects [13], [14], but to depict the development of functional networks at different life stages [15]–[17] and to investigate the pathologies of various diseases such as Alzheimer’s disease [18]–[20], schizophrenia [21], [22], depression [23] and multiple sclerosis [24].

Despite its relatively widespread use, the potential of ICA in RS-fMRI data analysis is not always realized. In most applications, the spatial patterns of the acquired RSNs are the primary focus, and the differences in the spatial pattern of one or more RSNs between different subject groups are then identified [18]–[20], [22]–[24]. One exception is Garrity *et al.* [21], who analyzed not only the abnormalities in the spatial maps, but the frequency distribution of the time series of the default mode component in schizophrenia. The pioneering study by Garrity *et al.* indicated the potential use of the timeseries of separate RSNs acquired by ICA in evaluating brain functions [21]. Other investigations of the *temporal* characteristics of RSNs uniformly derived the RSN timeseries from predefined ROIs rather than ICA [25]–[27]. In these studies, the temporal correlation matrix (between ROIs or RSNs) was the measure of interest and was used to discriminate between disease state, brain state and age, respectively. Such studies indicated that both the temporal features of separate RSNs and the temporal correlation between RSNs can provide additional information besides those provided by spatial maps.

The aim of the study was to systematically investigate which features in ICA of resting-state fMRI relate to behaviour, with stop signal reaction time (SSRT) in a stop signal task taken as a test case. The stop-signal task has been shown to be a valuable tool for the study of response inhibition, a key component of executive control, and the SSRT measures subjects’ ability to inhibit a prepotent response. The study was carried out as follows: (1) Temporal-concatenation group-ICA was performed on the RS-fMRI data of 34 subjects to acquire the group independent component maps. The dataset has formerly been used in a study investigating the relationship between the regional homogeneity of RS-fMRI signals and individual performance in response inhibition, as evaluated by SSRT [28]. Dual regression was then performed to find subject-specific versions of the group maps, along with associated timeseries (for each RSN, of each subject); (2) RSN-SSRT relationships were analyzed from three perspectives. First, the standard deviation of each RSN timeseries was used to represent the strength (amplitude) of the RSN, and correlation between this amplitude and SSRT across subjects was performed for each RSN. Second, temporal correlations between two RSN timeseries were calculated, for every pair of RSNs and for every subject; these correlations were then correlated with SSRT across subjects. Finally, as has been done in most previous ICA-based analyses, SSRT was correlated with each spatial map in a voxel-wise way to evaluate the relationship between the RSN spatial maps and SSRT.

The influences of certain processing steps (on the final RSN-SSRT correlation results) were also evaluated in this study, and these included: (1) the influence of ICA decomposition dimension on the RSN-SSRT correlation results - all analyses were based on group-ICA decompositions at two dimensionalities, namely, 27 dimensions (the optimal dimensionality chosen by the ICA algorithm) and 70 dimensions [14]; (2) the influence of different correlation-matrix estimation techniques, including full (normal) correlation and partial correlation [29]; (3) the influence of z-transformation and spatial smoothing of the spatial maps.

## Materials and Methods

### Ethics Statement

Written informed consent was obtained from each participant, and the study was approved by the Institutional Review Board of the State Key Laboratory for Cognitive Neuroscience and Learning, Beijing Normal University.

### Subjects

Thirty-four healthy male subjects (23.7±3.8 years) participated in the present study. All were right-handed and had no history of neurological or psychiatric disorders. For each subject, we acquired both MRI data and performance data in the stop-signal task. Each subject was required to perform stop-signal tasks outside the scanner *immediately after* scanning.

### Behavioral Data Acquisition

Each subject performed a stop-signal task including 240 Go trials and 60 Stop trials. For the Go trials, subjects were instructed to respond as quickly as possible without sacrificing accuracy by clicking the mouse button according to the Go signal that appeared on the centre of the computer screen. For the Stop trials, subjects were instructed to stop their response on seeing a stop signal. By dynamically adjusting the time interval between the Stop and Go signals (stop-signal delay, SSD) of each Stop trial, approximately 50% successful response inhibition was yielded. Each subject’s ability in response inhibition, as measured by the stop-signal reaction time (SSRT), was estimated by subtracting SSD from the mean value of reaction time (RT) of Go trials [30], [31]. More details can be found in the paper by Tian *et al.* [28].

### MRI Data Acquisition

MRI data were obtained using a 3.0 Tesla Siemens Trio scanner at the Imaging Center for Brain Research, Beijing Normal University. Since the RS-fMRI signal can be sensitive to preceding events [32], each subject underwent an 8-min fMRI scan during a conscious resting state immediately after the acquisition of the localizer images. Functional images were collected axially using an echo-planar imaging sequence sensitive to blood oxygen level dependent (BOLD) contrast with the following parameters: 33 slices, 2,000/30 ms (TR/TE), 3.5/0.7 mm (thickness/gap), 220×220 mm (FOV), 64×64 (resolution), 90^o^ (flip angle). During the resting state, the subjects were instructed to remain still, awake with their eyes closed, as motionless as possible and to think of nothing in particular (none of the subjects fell asleep during scanning, according to a simple questionnaire administered immediately after the scan). Whole brain 3D T1-weighted images were then obtained sagittally with the following parameters: 128 slices, 2,530/3.39 ms (TR/TE), 1.33/0 mm (thickness/gap), 256×256 (resolution), 240×240 mm (FOV), 7^o^ (flip angle).

### RS-fMRI Data Preprocessing

RS-fMRI data preprocessing was performed using the FMRIB Software Library (FSL) [33], [34]. The following processing steps were applied to the RS-fMRI data of each subject: (1) Removal of the first 10 volumes; (2) Correction for head motion with Motion Correction using FMRIB’s Linear Image Registration Tool (MCFLIRT) [35]; (3) Removal of non-brain tissues with Brain Extraction Tool (BET) [36]; (4) Spatial smoothing using a Gaussian kernel of full width at half maximum (FWHM) 5 mm; (5) Removal of slow drift by high-pass temporal filtering (cutoff period = 100.0 s); (6) Registration of the subject’s RS-fMRI data to their high-resolution structural image and then to MNI152 standard space using FMRIB’s Linear Image Registration Tool (FLIRT) and FMRIB’s Nonlinear Image Registration Tool (FNIRT) [35], [37], and resampling of the subject’s registered RS-fMRI data in MNI152 space to 2×2×2 mm resolution. Given that effects associated with head motion and other artifactual sources are ameliorated through the use of ICA and multiple-regression within the dual-regression, and artefactual ICA components later removed, no denoising was performed here.

### Group-ICA and Dual-Regression

Temporal-concatenation group-ICA was carried out using Multivariate Exploratory Linear Optimized Decomposition into Independent Components (MELODIC) tool in FSL [38]. ICA was performed at two dimensionalities *d*: one was estimated automatically by the MELODIC software (giving *d* = 27), and another was set to *d* = 70. All following analyses were based on the group-ICA results from both dimensionalities.

Dual regression was then applied to each subject’s preprocessed RS-fMRI data to build subject-level versions of the group-ICA maps, and associated timeseries [39], [40]. Specifically, *for each subject* (separately): (1) the formerly obtained *d* group-ICA spatial maps (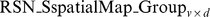, with *v* indicating the number of voxels in a spatial map, and here *v* = 91×109×91) were used as spatial regressors against the subject’s 4D RS-fMRI data (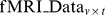, with *t* indicating the number of volumes, and here t = 230), to estimate the RSN timeseries (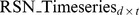) based on:
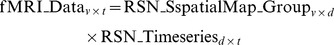
(1)


Before being fed into the model, each component map was demeaned and normalized. The equation is solved by pre-multiplying both sides by the pseudo-inverse of the group maps matrix. (2) The resulting *d* RSN timeseries were then used as temporal regressors against each subject’s preprocessed 4D RS-fMRI data, to estimate the *d* individual-subject-level spatial maps (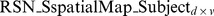) based on:
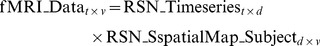
(2)


Again, each RSN timeseries was demeaned and normalized before entering into the regression model.

Among the *d* RSNs, some are functionally interpretable and some were judged to be associated with artifactual sources, such as head motion, cerebral spinal fluid pulsation, white matter or large blood vessels. Therefore, before evaluating the relationship between SSRT and components, *d’* functionally interpretable components were selected by visual inspection. The analyses of the RSN-SSRT relationships were only based on these *d’* functionally interpretable components.

### RSN-SSRT Relationship Analyses

We analyzed RSN-SSRT correlations from three perspectives. First, the RSN timeseries standard deviation (calculated *before* it was normalized for use as the regressor in the second dual regression stage) was estimated, to represent the strength (amplitude) of each component in each subject. We then evaluated the amplitude-vs-SSRT correlation, for each functionally interpretable component. The amplitude-vs-SSRT correlation results were thresholded at *p*<0.05 (FDR corrected, using *fdr* command in FSL).

Secondly, we estimated correlations between all pairs of the *d’* RSN timeseries (forming a network matrix of correlations). We then estimated the correlation between these values and SSRT, across subjects. The *d’*×*d’* “network matrices” (of correlations) were estimated in three ways, namely, the full correlation (CORR), the regularized normalized inverse of the covariance matrix (ICOV) [29], [41]–[44], using a lambda of 10 (Fig. S1, ICOV matrices evaluated based on lambdas of 5, 10 and 20 are generally larger in magnitude and less sparse than those based on 50, 100, and 200, and here we chose the middle, lambda = 10), and the inverse covariance (group-level covariance modeling) (gICOV) [45]. CORR evaluates the similarity between two timeseries directly, and reflects both *direct* and *indirect* functional connections [46]. Both ICOV and gICOV evaluate the similarity between two timeseries after regressing out the influences from all other timeseries, and are regularized (mathematically better conditioned) versions of partial correlation; these measures should emphasize *direct* functional connections, rather than *indirect* [29]. Whereas ICOV only regularizes the partial correlation matrix *within* subject (shrinking small/noisy estimates towards zero), gICOV additionally regularizes the matrices *across* subjects, to further ameliorate estimability problems. To diminish the influence of artifacts, the timeseries of the artifactual components were included in the ICOV and gICOV analyses (but matrix elements involving those were discarded at the point of investigating correlations against SSRT). The correlation/network matrices evaluated by the three methods were transformed into z-scores using the Fisher transform to improve normality, and the z-transformed correlations were then fed into SSRT correlation analyses. As almost no SSRT correlations survived a corrected threshold of *p*<0.05 (FDR corrected), to provide more qualitative results, we also report SSRT correlations at a more liberal threshold of *p*<0.01 (uncorrected).

Finally, voxel-wise correlation was performed between SSRT and the spatial-maps of the RSNs (the outputs of the dual regression). For each of the *d’* functionally interpretable RSNs, the individual-subject-level spatial map was first collected across subjects into a 4D file (the fourth dimension was subject number), and 5,000 permutations were performed on this 4D file to test for significant spatial-map-vs-SSRT correlations. The results were thresholded at p<0.05 (using threshold-free cluster enhancement (TFCE) in FSL [47], FWE corrected). Here, the effects of two processing steps were evaluated. One was the effect of the z-transformation of the spatial maps by dividing the relevant component weight by the standard deviation of the background noise (the residuals from the multiple-regression that forms the second stage of the dual regression). The distinction is equivalent to the difference between parameter estimates (betas or contrasts of betas), in general linear modeling, and Z-statistic maps of statistical significance. We performed SSRT correlations on both the spatial maps produced directly by dual-regression, and the z-transformed version of these spatial maps. Another factor evaluated was the effect of spatial smoothness, and here we evaluated SSRT correlations based both on the “unsmoothed” (i.e., only the 5-mm FWHM smoothing at the preprocessing stage) spatial maps and on the spatial maps additionally smoothed with a Gaussian kernel of FWHM 10 mm.

## Results

### Group-ICA Spatial Maps

**Figure 1:**
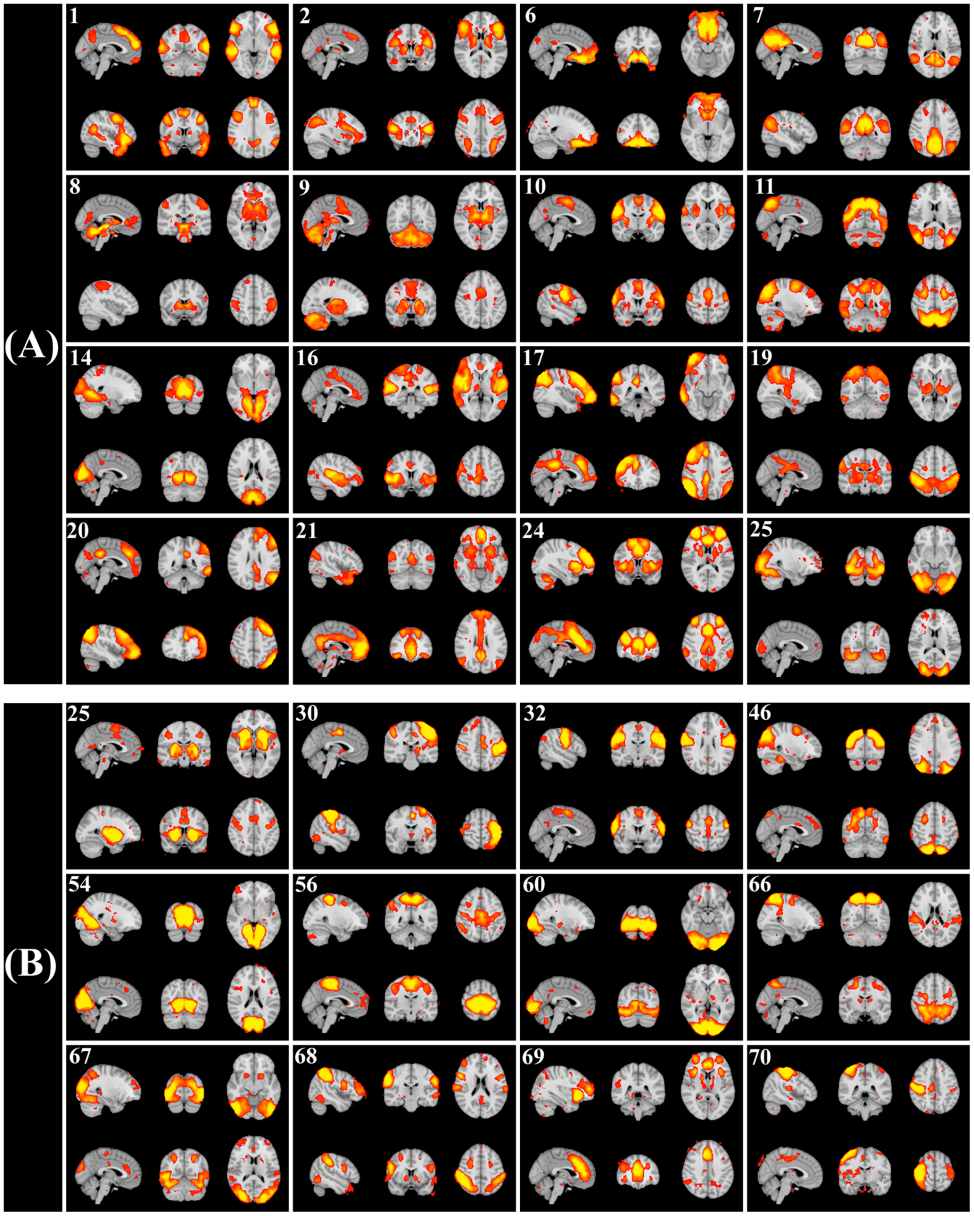
Group-ICA estimated RSNs based on 27-component analysis (A) and 70-component analysis (B). All sixteen non-artefactual components from the 27-component analysis (A) and 12 non-artefactual components exhibiting significant or marginal significant amplitude-vs-SSRT correlations from the 70-component analysis (B) were shown. The number of each component was based on the ranking of variance explained by the component. A summary of the functions of the components shown in subfigure (A) can be found in Table S1.

From the 27-component group-ICA analysis, 16 components were judged to be non-artefactual, based on visual inspection of each component’s spatial map. Spatial maps of these are shown in Fig. 1 (A). The functions and anatomy of these RSNs are summarized in Table S1 and further description of each of these RSNs can be found in Text S1. Maps of eleven RSNs judged to be artefactual can be found in Fig. S2.

Based on the 70-component analysis, 34 components were non-artefactual. Maps of those RSNs that exhibited significant amplitude-vs-SSRT correlation can be found in Fig. 1 (B), and the spatial maps of all other components can be found in Figs. S3, S4, S5, S6 and S7. As has been reported by Smith *et al.* [14], 70-component analysis provided a more detailed separation of functional networks than did the 27-component results. For instance, the motor network (IC No. 10) based on 27-component analysis was divided into 4 subnetworks (IC No. 30, 32, 56, 70) in the 70-component analysis, and the primary-occipital/higher visual network (IC No. 25) in the 27-component results was further divided into 3 subnetworks (IC No. 46, 60, 67) in the 70-component analysis (Fig. 1 (B)).

### Correlation of SSRT vs. RSN Amplitude (RSN Timeseries Standard Deviation) across Subjects

Six components from the 27-component analysis exhibited significant negative correlations between RSN timeseries standard deviation and SSRT, at a threshold of *p*<0.05 (FDR corrected) (Table 1, Fig. S8). For components based on the 70-component analysis, negative SSRT correlations from 9 components were significant at a threshold of *p*<0.05 (FDR corrected) (Table 1, Fig. S9). No significant *positive* SSRT correlation was observed for any RSN from either of the two dimensions. Multiple comparison correction was carried out because of the number of RSNs’ amplitudes tested for correlation with SSRT (*d’* = 16 and 34, respectively, for the two dimensionalities), and a further correction factor of two (because testing for positive and negative correlations) was applied here.

**Table 1:** Significant negative correlation of RSN amplitude (timeseries standard deviation) vs. SSRT.

27 dimensions			70 dimensions		
IC No.	r	p-value(uncorrected)	IC No.	r	p-value(uncorrected)
***Motor Network***					
10^a^	−0.37	0.031	30	−0.44	0.0089
			32	−0.44	0.0085
			70	−0.46	0.0069
			56	−0.43	0.011
***Motor Control Network***					
9	−0.47	0.0051	25^ a^	−0.41	0.016
***Visual Network***					
14	−0.51	0.0022	54	−0.51	0.0019
25	−0.47	0.0051	46	−0.52	0.0018
			60	−0.47	0.005
			67 ^a^	−0.37	0.033
***Dorsal attention network***					
11	−0.45	0.0069	68	−0.49	0.0032
19	−0.46	0.0064	66	−0.51	0.0018
***Task***−***activation network***					
24	−0.41	0.015	69^ a^	−0.40	0.018

The results obtained at two decomposing dimensions, arranged according to their correspondence in the function of components. The threshold (determining inclusion in this table) was *p*<0.05 (FDR corrected), which corresponds to uncorrected 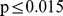 for the 27-component analysis and uncorrected 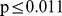 for the 70-component analysis. For components exhibiting significant SSRT correlations, their counterparts obtained at the other decomposing dimension were also listed here even if they did not survive the threshold, and are indicated by ^a^.

The results based on the two different decomposing dimensions correspond to each other well (Table 1). Specifically, clear correspondence was observed for the components corresponding to the motor control network, the primary-medial visual network, the two dorsal attention networks (DANs) (identified by the bilateral intraparietal sulcus and the bilateral frontal eye field) [48], [49] and the task-activation network (as proposed in [6], identified by lateral frontal-parietal regions, the dorsal anterior cingulate cortex (dACC) and the orbital-frontoinsula regions). All four sub-networks of the motor network based on 70-component analysis exhibited significant negative SSRT correlation; correlation of their counterpart from the 27-component analysis only survived a less strict threshold of *p*<0.05 (uncorrected). For the primary-occipital/higher visual network, the one component from the 27-component analysis and two of the three components from the 70-component analysis exhibited significant amplitude-SSRT correlations (*p*<0.05, FDR corrected), and correlation of the third visual component only survived a threshold of *p*<0.05 (uncorrected).

### Temporal Correlation between RSNs, and the Correlation of This with SSRT across Subjects

**Figure 2:**
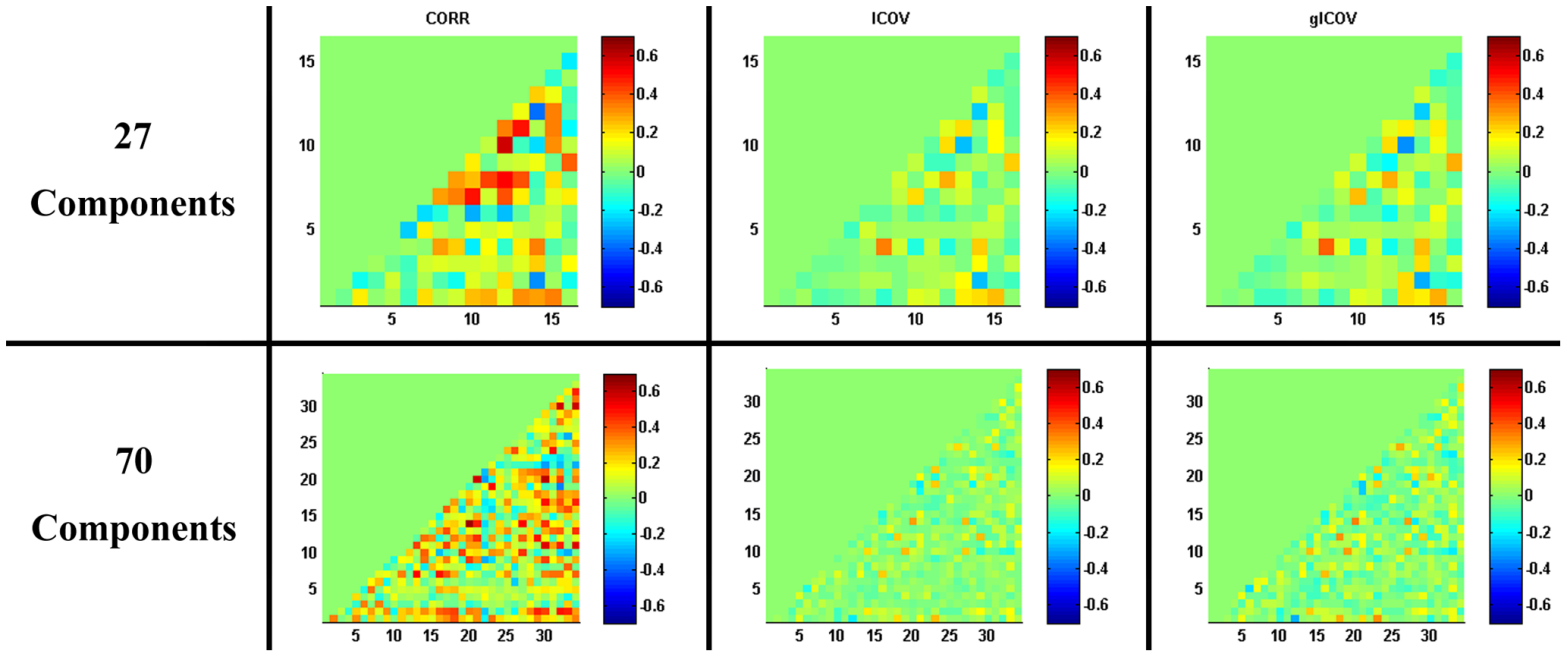
Maps of the mean (across subjects) full/partial correlation matrices. The coordinates in each subfigure indicate the number of the component within the set of non-artefactual components. The correspondence between the component numbers and their coordinates in the subfigures can be found in Table S2.

Fig. 2 shows the mean (across subjects) full/partial correlation matrices evaluated by the 3 correlation-matrix methods. It can be seen that the results look somewhat similar, specifically, the results based on the two partial-correlation-oriented methods (ICOV & gICOV) are very similar, and the correlations evaluated by CORR are generally larger in magnitude (and less sparse) than their counterparts based on partial-correlation methods (as expected).

Temporal correlations between components are of functional significance. For instance, based on 27-component analysis, strong *positive* correlations were observed between the two visual networks (Coordinate 9, IC No. 14 and Coordinate 16, IC No. 25), between the two default mode components (DMN) (Coordinate 4, IC No. 7 and Coordinate 14, IC No. 21) and between the two DANs (IC No. 12 and IC No. 8), although in the latter two cases this may be partly driven by spatial blurring and induced spatial overlap between strongly neighbouring components (this is less likely in the visual case). Strong negative correlations were observed between the anterior DMN (Coordinate 14, IC No. 21) and the two DANs (Coordinate 8, IC No. 11; Coordinate 12, IC No. 19), and between the posterior DMN (Coordinate 4, IC No. 7) and the secondary DAN (Coordinate 12, IC No. 19). However, the correlation between the posterior DMN (Coordinate 4, IC No. 7) and the primary DAN (Coordinate 8, IC No. 11) was positive.

**Figure 3:**
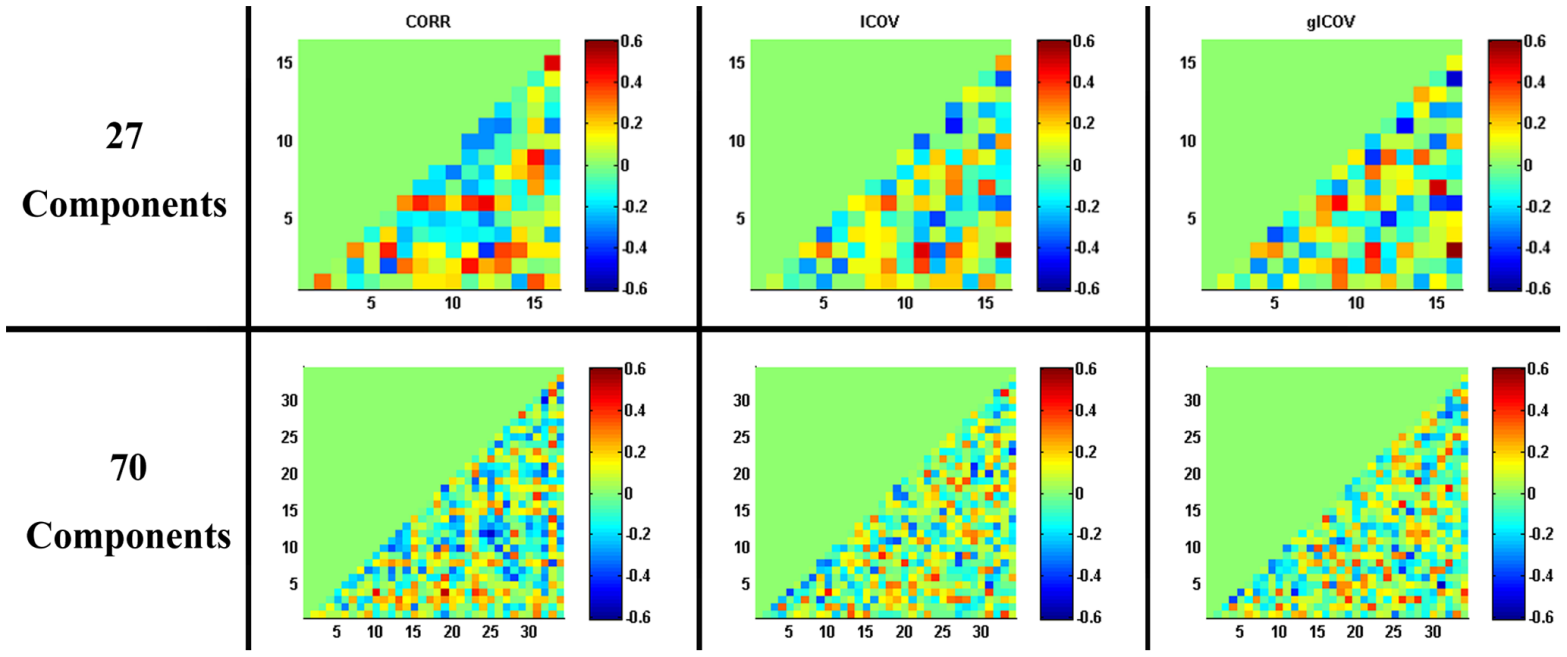
Correlation of SSRT vs. the full/partial (within-subject) RSN timeseries correlation matrices across subjects.

The SSRT correlations based on the RSN timeseries correlation *r* values and z-transformed-*r* values were very similar. Therefore, we report only the results based on z-transformed correlation matrices here. Fig. 3 shows the SSRT correlations across subjects. The results based on the two partial-correlation methods (ICOV & gICOV) were very similar (see also the right column of Fig. S10). The results based on full correlation methods are more different, but still bear some resemblance to the partial-correlation results (see also the left and middle columns of Fig. S10).

From the 27-component analysis, gICOV gave significant correlation with SSRT (*p*<0.05, FDR corrected) for just one network matrix element (i.e., only one pair of RSN timeseries), and no significant SSRT correlation was found when using the CORR and ICOV methods. This single pair of RSNs, showing a positive temporal partial-correlation that covaried positively with SSRT, is Nos. 6 and 25, the orbital-frontal and primary-occipital/higher visual networks (see the third row and the second column of Fig. S11). From the 70-component analysis, no significant SSRT correlation was observed from any of the three methods. To provide a qualitative view of the distribution of the reasonably strong SSRT correlations, we report results based on the 27-component analysis that survived a threshold of uncorrected p<0.01 (Table 2, see also Fig. S11).

**Table 2:** Network matrix vs. SSRT correlations from the 27-component analysis that survived a threshold of uncorrected *p*<0.01.

Component No.		CORR		ICOV		gICOV	
IC_1_	IC_2_	r-value	p-value (uncorrected)	r-value	p-value (uncorrected)	r-value	p-value (uncorrected)
24	25	**0.48**	0.0042	0.26	0.14	0.13	0.47
9	19	**0.46**	0.0064	0.083	0.64	0.099	0.58
2	17	**0.44**	0.0096	0.37	0.032	0.36	0.036
9	14	0.32	0.066	0.35	0.040	**0.45**	0.0071
10	24	0.26	0.14	0.35	0.044	**0.50**	0.0026
6	25	0.17	0.34	**0.53**	0.0013	**0.59** *	0.00024
6	17	0.15	0.41	**0.48**	0.0037	0.43	0.011
21	25	0.12	0.48	−0.37	0.031	−**0.53**	0.0014
17	20	−0.31	0.074	−**0.45**	0.0081	−**0.47**	0.0053

* indicated that the SSRT correlation survived a threshold of *p*<0.05 (FDR corrected). R-values in bold indicate that the SSRT correlation survived the threshold of uncorrected *p*<0.01.

### Correlation of SSRT vs. RSN Spatial Maps across Subjects

**Figure 4:**
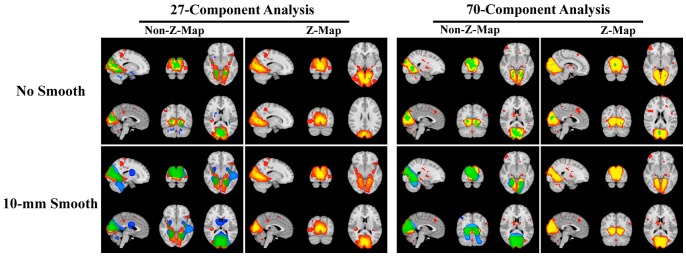
The effects of spatial smoothing and z-transformation upon spatial-map-vs-SSRT correlations. The *negative* spatial-map-vs-SSRT correlation maps were thresholded at *p*<0.05 (TFCE corrected for multiple comparisons across space, and for two-sided tests, but not corrected across multiple RSNs). *Non-Z-Map* indicates SSRT correlations on the spatial maps produced directly by dual-regression, and *Z-Map* indicates the correlations produced by the z-transformed version of these spatial maps. *No Smooth* indicates SSRT correlations based on the unsmoothed (i.e., only the 5-mm FWHM smoothing at the preprocessing stage) spatial maps, and *10-mm smooth* indicates the SSRT correlations based on the spatial maps additionally smoothed with a Gaussian kernel of FWHM 10 mm. The correlation maps were superimposed on their respective *group-mean* spatial maps obtained by Group-ICA and then on the MNI152 template. The *group-mean* spatial maps were provided here to show whether the significant regions lie within or outside the group-level RSNs. Red-yellow indicates significant regions in the *group-mean* spatial map, blue indicates significant spatial-map-vs-SSRT correlations that do not overlap with the group-mean map, and green indicates regions of overlap. The results were based on components corresponding to primary-medial (high eccentricity) visual networks, namely, component No. 14 from the 27-component analysis and component No. 54 from the 70-component analysis. The spatial-map-vs-SSRT correlations based on the z-transformed and 10-mm smoothed components were also shown though no significant voxel was observed. It can be seen that a greater number of significant voxels, if any, could be detected based on non-z-transformed and 10-mm smoothed spatial maps.

Results relating to the influences of z-transformation and spatial smoothing on the spatial-map-vs-SSRT correlations are provided in Table 3, Figs. 4 and and S12. Significant spatial-map-vs-SSRT correlations were observed in regions within the medial and lateral visual networks, the motor network, the two DANs and the DMN (Fig. 4, S11, Table 3). In addition, significant correlations were also found in the dACC on the spatial-map of the motor network (see subfigures 70-70-N-S and 70-70-Z-S of Fig. S12), and the visual regions on the spatial-map of the secondary DAN (see subfigure 70-66-N-S of Fig. S12).

**Table 3:** The effects of z-transformation and spatial smoothing on spatial-map-vs-SSRT correlations.

27-Component Analysis							
IC No	Smoothed	N_z_	N_non-z_	N_overlap_	P_overlap_ (%)	TFCE enhanced, FWE corrected	
						min *p_z_*	min *p_non−z_*
14	N	35^a^	7888	0	0.00	0.030	0.0032
	Y	–	33885	–	–	–	0.004
16	N	–	18	–	–	–	0.036
	S	–	37	–	–	–	0.050
19	N	–	15^a^	–	–	–	0.022
25	N	–	2217	–	–	–	0.0068
	S	–	92	–	–	–	0.049
**70-Component Analysis**							
IC No	Smoothed	N_z_	N_non-z_	N_overlap_	P_overlap_ (%)	TFCE enhanced, FWE corrected	
						min *p_z_*	min *p_non−z_*
29	Y	2853	3804	2597	91.03	0.029	0.022
36	N	–	17^a^	–	–	–	0.030
54	N	173^a^	4782	173	100.00	0.010	0.0008*
	Y	–	18296	–	–	–	0.0010*
56	N	–	507	–	–	–	0.0092
	Y	–	4551	–	–	–	0.0156
60	N	295	2714	292	98.98	0.008	0.004
	Y	6390	28635	6390	100.00	0.026	0.0116
66	N	–	1067	–	–	–	0.0024
	Y	–	32157	–	–	–	0.002
70	N	–	1751	–	–	–	0.0036
	Y	2426	60624	2426	100.00	0.0016	0.0008*

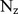
 is the number of significant voxels based on z-transformed spatial maps; 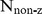 is the number of significant voxels based on original spatial maps; 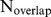 is the number of significant voxels in common for the results based on z-transformed spatial maps and those based on original spatial maps; 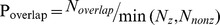 describes the consistency of the results based on z-transformed spatial maps and those based on original spatial maps. The threshold was p<0.05 (TFCE enhanced, FWE corrected across space, two-sided, but not corrected across RSNs). 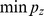 is the TFCE corrected p-value (across space, two-sided, but not across RSNs) of the strongest spatial-map-vs-SSRT correlation based on z-transformed spatial maps, and 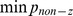 is that based on original spatial maps. “Y” indicates the results were based on 10-mm smoothed spatial maps and “N” indicates no spatial smoothing. * indicates that the SSRT correlation survived a fully-corrected threshold of p<0.05 (TFCE enhanced, FWE corrected for multiple comparisons across space, and for two-sided tests, and further corrected across multiple RSNs. To note, these RSNs also showed significant amplitude-vs-SSRT correlations). a indicates that significant clusters were detected only on the correlation maps based on unsmoothed spatial-maps.

Not surprisingly, spatial smoothing of the spatial maps increased the spatial smoothness of the spatial-map-vs-SSRT correlation maps (Fig. 4). Moreover, a greater number of significant voxels (*p*<0.05, TFCE enhanced, FWE corrected for multiple comparisons across space, and for two-sided tests, but not corrected across multiple RSNs), if any, were detected on the correlation maps based on 10-mm smoothed spatial maps, compared to those based on unsmoothed spatial maps (Table 3). A greater number of significant voxels were observed based on the original spatial maps as compared to those based on the z-transformed spatial maps (Table 3). This was true for all 7 cases (showing significant results) based on the 27-component analysis and all 12 cases based on the 70-component analysis. The spatial-map-vs-SSRT correlations based on the original (BOLD amplitude) and z-transformed spatial-maps were largely overlapping. In five out of six cases, more than 90 percent of significant voxels based on z-transformed spatial maps lay *within* (group mean) significant clusters acquired based on original spatial maps (Table 3).

It should be noted that the present spatial-map-vs-SSRT correlations were performed on each of the non-artefactual spatial maps separately. For full consideration of multiple comparisons, more stringent correction *should* be applied. If Bonferroni correction were adopted here, then an appropriate threshold should be *p (already corrected over space and two-tailed testing)* <0.0031 (0.05/16) for the 27-component analysis, and *p*<0.0015 (0.05/34) for the 70-component analysis. As can be seen from Table 3, none of the eight “significant” results based on the 27-component analysis and only three of the seventeen “significant” results based on the 70-component analysis survived this more stringent thresholding.

## Discussion

In this study, we systematically investigated which features in ICA of resting-state fMRI relate to subjects’ behaviour, with their performance in response inhibition measured by SSRT taken as a test case. For multiple networks, we found significant (fully corrected) correlations not only between SSRT and spatial maps, but also between SSRT and RSN timeseries amplitude. Most of these amplitude- and spatial-map-vs-SSRT correlations were of functional interpretability. The temporal correlations between RSN pairs were of functional significance, but correlation-network-matrix methods tested did not appear to be very sensitive to finding SSRT correlations. A detailed discussion of the methods and results are as follows.

### Multiple Networks Were Found to Relate to Individual Differences in SSRT When Judging by RSN Timeseries Amplitude

SSRT correlations were performed on each component in this study to investigate the relationship between the “strength” of a component *as a whole* and SSRT. The results showed that the SSRT correlations based on the 27-component analysis and those based on the 70-component analysis correspond to each other well. This correspondence between the results based on different decomposition dimensions indicated that exact decomposition dimension would hopefully have limited impact on the RSN timeseries amplitude-based results.

In this study, only negative amplitude-vs-SSRT cross-subject correlations were observed, indicating that higher amplitudes of the RSNs are associated with better performance in inhibition, as evaluated by shorter SSRT. The RSNs exhibiting significant SSRT correlations include not only networks for sensory-motor processing, including the medial and lateral visual networks, the motor and the motor control networks, but also networks specialized for higher-level cognitive processing, including the DAN/task-activation network (Table 1, Figs. S8 and S9).

Most of these significant amplitude-vs-SSRT correlations are of functional interpretability. For instance, the significant correlations observed in the motor and motor control networks in this study might also be interpreted by their own roles in response inhibition, as components of these two networks have been reported to play critical roles in inhibition [50], [51]. The present finding of significant amplitude-vs-SSRT correlations in the visual networks based on RS-fMRI are consistent with the findings of significant activation-vs-SSRT correlations based on a stop-signal task with visual stimulus (see Fig. 2C in [52] and Fig. 3B in [53]). The DAN has been deemed to “link relevant sensory representations to relevant motor maps” [49]. The task-activation regions have frequently been reported to coactivate in response to error, attention and response selection [54]–[56], which are closely related to response inhibition [50]. Menon *et al.* reported coactivation of the task-activation regions in a Go/No-Go task, another paradigm in which response inhibition is involved [54]. The present finding of significant SSRT correlation in the DAN and task-activation network is consistent with these former findings.

### Temporal Correlations between RSNs Were of Functional Significance, but Were Not Very Sensitive to Finding SSRT Correlations

Three network (correlation) matrix evaluation techniques were used in this study to evaluate the strength of the interactions between RSNs. The group-mean matrices acquired by different evaluation techniques were reasonably similar, with stronger and less sparse correlations observed via the CORR (full correlation) method (Fig. 2). This is expected, because partial-correlation methods aim to remove mutual dependencies originating from other brain regions’ common influences [57].

The temporal correlations between RSN pairs were of functional significance. For instance, based on 27-component analysis, strong positive correlations were observed between the medial and lateral visual networks, between the anterior and posterior parts of the DMN and between the two DANs. These findings are consistent with former findings of close relations among visual regions [58], DMN regions [3] and DAN regions [48]. The DMN, as a whole, has formerly been reported to be negatively correlated with the so-called task-positive network [59], most of which overlaps the present DANs. In the present study, strong negative correlations were observed between the anterior DMN and the two DANs, and between the posterior DMN and the secondary DAN, a result consistent with previous work based on seed-based correlation [59]. However, the correlation between the posterior DMN and the primary DAN was positive in this study. Moreover, the positive correlation between the two networks evaluated by the partial-correlation methods was the strongest among the 120 possible component-pairs based on 27-component analysis. This finding might be explained by the functional heterogeneity of the anterior and posterior parts of the DMN [60]. The functional significance of the strong positive correlation between the posterior DMN and the primary DAN needs to be further investigated.

In contrast to RSN amplitude-vs-SSRT correlations, almost no SSRT correlations with network matrices survived thresholding at *p*<0.05 (FDR corrected) (Table 2, Fig. 3). Here the number of multiple comparisons to correct for is much higher than when correlating against RSN amplitude: 120/561 two-tailed comparisons for the 27−/70-component analyses, respectively, compared to 16/34 comparisons for amplitude-vs-SSRT correlations. Therefore, some relatively strong network-matrix-vs-SSRT correlations did not survive the FDR corrected thresholding, even if the strength of these correlations were comparable to those significant amplitude-vs-SSRT correlations (i.e., r >0.4, see Table 2).

### RSN Spatial-Map-vs-SSRT Correlations Provided Additional Information about RSN-SSRT Relationships

The effects of increased spatial smoothing and z-transformation of the RSN spatial maps on the spatial-map-vs-SSRT correlations were evaluated. In a recent resting state fMRI study by Wu *et al.* [61], spatial smoothing has been reported to consistently increase spatial extents of seed-based correlations. In the current study, for all cases when significant clusters were detected based on smoothed spatial maps, the significant clusters were larger than those based on unsmoothed spatial maps (Table 3, Figs. 4 and S12). This could be expected because spatial smoothing can enhance signal to noise ratio, and possibly ameliorate imperfect functional alignment between subjects, and thus would increase the sensitivity of SSRT correlation analysis, as long as the signal of interest is not spatially too small or finely detailed. However, there were 4 cases where (small) significant clusters were detected only on the correlation maps based on *unsmoothed* spatial-maps (Table 3).

Z-transformation uniformly lowered the sensitivity of detecting significant SSRT correlations. Specifically, for all 7 cases based on 27-component analysis and all 12 cases based on 70-component analysis, more significant voxels were detected based on the “original” RSN spatial maps (meaning the size of the regression parameter of the BOLD signal against the normalized RSN timeseries), as compared to those based on the z-transformed spatial maps. Z-transformation was carried out by dividing original BOLD regression map by the standard deviation of the background noise, and thus the Z-transformed spatial maps measure the RSN’s (within-subject) signal-to-noise ratio [62], while the original spatial maps acquired directly from dual-regression analysis reflect just the RSN’s BOLD amplitude (and spatial coherence). The present results indicate that including the background noise information into the spatial maps did not benefit brain-behaviour relationship analysis.

Spatial-map-vs-SSRT correlations were performed to evaluate the relationship between SSRT and involvement of different brain regions in the RSNs. The medial and lateral visual networks, the motor network, and the two DANs exhibited significant amplitude-SSRT correlations (Table 1), and here, significant spatial-map-vs-SSRT correlations were observed in regions within these networks (Fig. 4 and S12, Table 3). These results not only supported the importance of these networks for individual performance in inhibition evaluated by SSRT, but indicated that some features of brain function, found through investigation of the RSN timeseries, can also be detected based on spatial maps, though in our case with less statistical sensitivity, when fully correcting for multiple comparisons over space and RSNs tested.

Some significant SSRT correlations found through investigation of the spatial maps might well be unreported if only the RSN timeseries are investigated. For instance, significant spatial-map-vs-SSRT correlations were also detected with respect to the involvement of two regions of the DMN with the rest of the DMN, namely, the right inferior parietal lobue and the posterior cingulate cortex (see subfigures 70-29-N-S and 70-29-Z-S of Fig. S12). Despite the interactions between networks and regions within themselves, the interaction between the dorsal anterior cingulate cortex (dACC) and the motor network (see subfigures 70-70-N-S and 70-70-Z-S of Fig. S12), as well as that between the visual regions and secondary DAN (see subfigures 70-66-N-S of Fig. S12), were also observed to be related to individual differences in response inhibition. A discussion of the functional significance of these correlations can be found in Text S2.

### Multiple Comparison Correction Issue

The problem of multiple comparison correction becomes greater, with greater numbers of amplitudes/correlations/voxels tested, and hence the less “rich” measures suffer the least from this, and hence may be the most likely to survive fully corrected thresholding. Consistent with this common sense, the amplitude-vs-SSRT correlations of several networks were fully correctable because of relatively fewer comparisons (16/34 comparisons for 27−/70-component analysis), and nearly no network-matrix-vs-SSRT correlations survive the FDR corrected thresholding because of more comparisons, even if the strength of these correlations were comparable to those significant amplitude-vs-SSRT correlations (i.e., r >0.4, see Table 2). The spatial-map-vs-SSRT correlations discussed above were also based on a relatively loose threshold, which considered only the corrections for the number of voxels and the positive and negative correlations, and did not further consider the number of RSNs. For full consideration of multiple comparisons, more stringent correction *should* be applied. If Bonferroni correction were adopted, none of the eight “significant” results based on the 27-component analysis and only three of the seventeen “significant” results based on the 70-component analysis survived this more stringent thresholding.

Because of a greater number of comparisons, statistics based on spatial-maps and network-matrices are less likely to survive a fully corrected threshold compared with RSN amplitude. If, instead of the above, a post-hoc analysis was applied to test only the spatial maps of the RSNs for which the RSN timeseries amplitude already showed a significant SSRT correlation, more “significant” results found would survive this correction (for the number of RSNs’ spatial maps tested); of course, this is not strictly valid (being a somewhat circular analysis), but, in general, if one has a truly a priori reason to only consider one RSN (or a few RSNs) in the final cross-subject analyses, the problem of the multiple-RSNs-comparison-correction will be alleviated.

All tests carried out in this study were univariate, meaning that each single element in each different kind of measure (i.e., each different RSN’s time series amplitude, each different correlation matrix element, or each voxel in an RSN spatial map) was tested for correlation against SSRT, largely independently of each other element (though voxels in the spatial map are enhanced in terms of their cluster-like behavior through the use of TFCE, and hence this test is weakly multivariate). We chose to do this in order that the results based on each measure (RSN amplitude, correlation between RSN pairs, and RSN spatial-map) would be as clear and interpretable as possible, but we note that the univariate approach does not expect to maximize overall sensitivity to finding some correlation against the “non-imaging” variable (in this case SSRT) – for that, one would move to multivariate machine learning approaches. Related to this, our correction for multiple comparisons across elements is not the (generally over-conservative) Bonferroni approach, but the more accurate empirical FWE method made possible through the use of permutation testing, generating the null distribution of the maximum test statistic across elements tested. The only case where this was not done was when considering the correction for multiple comparisons *across RSNs* in the case of spatial map testing, where we discussed either the use of full Bonferroni, or a weaker (and less valid) correction over only the RSNs showing a result in the RSN amplitude tests.

### Conclusions

In this study, we systemically investigated which features in ICA of resting-state fMRI relate to behaviour, with stop signal reaction time (SSRT) taken as a test case. The results indicate that the time series amplitudes of the motor and visual networks, as well as those of the DAN and task-activation network are correlated with SSRT. Spatial-map-vs-SSRT correlations could not only detect the influences of these networks upon SSRT, but find other networks, as well as interaction between brain regions and networks, important for individual performance in inhibition evaluated by SSRT. The temporal correlations between RSN pairs were of functional significance, but these correlations did not appear to be very sensitive to finding SSRT correlations. In summary, the temporal information acquired by ICA provided us a perspective complementary to that provided by spatial-maps. This complementary role would be helpful not only for the analyses of brain function in normal subjects, but also for the pathology analyses of psychiatric diseases by selecting candidate RSNs for further analyses based on the their temporal information. In addition, as compared to spatial maps, the temporal information of RSNs are of relatively low dimension and might be directly used to classify psychiatric patients and controls based on RS-fMRI, without necessarily performing feature dimensionality reduction.

## Supporting Information

Figure S1
**Maps of the mean (across subjects) ICOV matrices (Upper) and the ICOV-vs-SSRT correlations (Lower) acquired with different lambdas.** The coordinates in each subfigure indicate the number of the component within the set of non-artefactual components. The correspondence between the component numbers and their coordinates in the subfigures can be found in Table S2. 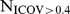 and 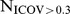
indicate the number of mean-ICOVs larger than 0.4 and 0.3, respectively. 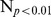 and 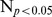 indicate the number of significant ICOV-vs-SSRT correlations at thresholds of p<0.01 (uncorrected) and p<0.05 (uncorrected), respectively. It can be seen that the mean ICOV matrices evaluated based on lambdas of 5, 10 and 20 are generally larger in magnitude and less sparse than those based on 50, 100, and 200. Based on 27-component analysis, more significant ICOV-vs-SSRT correlations were found with lambdas of 5, 10 and 20 (compared with 50, 100 and 200), and the strength of the correlations based on 70-component analysis were approximately the same between different lambdas. In this paper, results based on lambda = 10 were reported in detail.(TIF)

Figure S2 **Maps of 11 components regarded to be associated with artifactual sources from the 27-component analysis.** This figure shows every 3rd axial slice in 2-mm MNI152 standard space, starting with the lowest slice at −54 mm. On the right is the number of each component, which was based on the ranking of variance explained by the component.
(TIF)

Figure S3 **Maps of 11 of components from the 70-component analysis.** These components were considered non-artefactual, but not shown in Fig. 1(B) in the main text. This figure shows every 3rd axial slice in 2-mm MNI152 standard space, starting with the lowest slice at −54 mm. On the right is the number of each component, which was based on the ranking of variance explained by the component.
(TIF)

Figure S4 **Maps of a further 11 components from the 70-component analysis.** These components were considered non-artefactual, but not shown in Fig. 1(B) in main text. This figure shows every 3rd axial slice in 2-mm MNI152 standard space, starting with the lowest slice at −54 mm. On the right is the number of each component, which was based on the ranking of variance explained by the component.
(TIF)

Figure S5 **Maps of 12 of 36 components regarded to be associated with artifact sources from the 70-component analysis.** This figure shows every 3rd axial slice in 2-mm MNI152 standard space, starting with the lowest slice at −54 mm. On the right is the number of each component, which was based on the ranking of variance explained by the component.
(TIF)

Figure S6 **Maps of a further 12 of 36 components regarded to be associated with artifact sources from the 70-component analysis.** This figure shows every 3rd axial slice in 2-mm MNI152 standard space, starting with the lowest slice at −54 mm. On the right is the number of each component, which was based on the ranking of variance explained by the component.
(TIF)

Figure S7 **Maps of a further 12 of 36 components regarded to be associated with artifact sources from the 70-component analysis.** This figure shows every 3rd axial slice in 2-mm MNI152 standard space, starting with the lowest slice at −54 mm. On the right is the number of each component, which was based on the ranking of variance explained by the component.
(TIF)

Figure S8
**Plots of significant negative cross-subject correlation of RSN timeseries amplitude (standard deviation) vs. SSRT, based on 27-component analysis.** The threshold was *p*<0.05 (FDR corrected), which corresponds to uncorrected 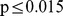. SSRT correlation of component No. 10, which did not survive thresholding, is also shown because its counterparts obtained at 70-dimensions exhibited significant SSRT correlation (see Table 1 in the main text for more details).(TIF)

Figure S9
**Plots of significant negative cross-subject correlation of RSN timeseries amplitude (standard deviation) vs. SSRT, based on 70-component analysis.** The threshold was *p*<0.05 (FDR corrected), which corresponds to uncorrected 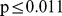. SSRT correlations of component Nos. 25, 67, 69, which did not survive thresholding, are also shown because their counterparts obtained at 27-dimension exhibited significant SSRT correlation (see Table 1 in the main text for more details).(TIF)

Figure S10 **Similarities between different methods for estimating RSN timerseries correlation matrices.** The correlations between SSRT and matrix elements is estimated for each method, and then compared between methods. It can be seen that the results based on the two partial-correlation methods (ICOV & gICOV) shared much resemblance to each other (the right column).
(TIF)

Figure S11 **SSRT and network matrix correlations surviving an uncorrected threshold of** ***p*** **<0.10 based on the 27-component analysis.** Only gICOV (6, 25) was fully significant (*p*<0.05, FDR corrected).
(TIF)

Figure S12 **Maps of other significant negative spatial-map-vs-SSRT correlations besides those shown in** **Fig. 4** **in the main text.** The threshold was *p*<0.05 (TFCE enhanced, FWE corrected). The correlation maps were superimposed on their respective *group-mean* spatial maps obtained by Group-ICA and then on the MNI152 template. The *group-mean* spatial maps were provided here to give illusive idea about whether the significant regions lie within or outside the RSNs. Red-yellow indicates significant regions in the group-mean RSN map, blue indicates significant spatial-map-vs-SSRT correlations, and green color indicates overlap between the two. Each subfigure was identified by an array of “Number-Number-Character-Character”. The first number of this array indicates the dimension of ICA decomposition; the second number indicates the component number; the first character indicates whether the spatial maps were z-transformed before spatial-map-vs-SSRT correlation: with “N” indicates NO z-transformation and “Z” indicates the opposite; the second character indicates whether the spatial maps were 10-mm spatially smoothed before spatial-map-vs-SSRT correlation: with “N” indicating NO spatial smoothing and “S” indicating the opposite. For instance, “27-16-N-N” indicates that the subfigure was based on the unsmoothed non-z-transformed spatial map of the component No. 16 based on 27-component analysis.
(TIF)

Table S1 Summary of each network from the 27-component analysis, and those exhibiting significant timeseries amplitude-vs-SSRT correlations from the 70-component analysis. The *IC No.* was based on the ranking of variance explained by the component.
(DOCX)

Table S2 Correspondence between the component numbers and their coordinates in the subfigures of Figs. 2 and 3 in the main text and Fig. S1. The *Component No.* is based on the ranking of variance explained by the component. The *Coordinate* indicates the number of the component within the set of non-artefactual components. This table bridges the *Component Nos.* used in other parts of the paper and the *Coordinates* in Figs. 2, 3 and S1. According to this table, for instance, the value at the coordinate (3, 14) in Fig. 2 based on 27 component-analysis indicates the mean correlation/partial-correlation between the components No. 6 and No. 21.
(DOCX)

Text S1 **Description of Each Non-Artefactual Component from the 27-Component Analysis.**
(DOCX)

Text S2 **A Discussion of the Functional Significance of Some Spatial-Map-vs-SSRT Correlations.**
(DOCX)
