# Supplementary figures and images for: Spatial vs. Temporal Features in ICA of Resting-State fMRI – A Quantitative and Qualitative Investigation in the Context of Response Inhibition
Source: PLoS One. 2013 Jun 18;8(6):e66572. doi: 10.1371/journal.pone.0066572 (PMC3688987; doi:10.1371/journal.pone.0066572)

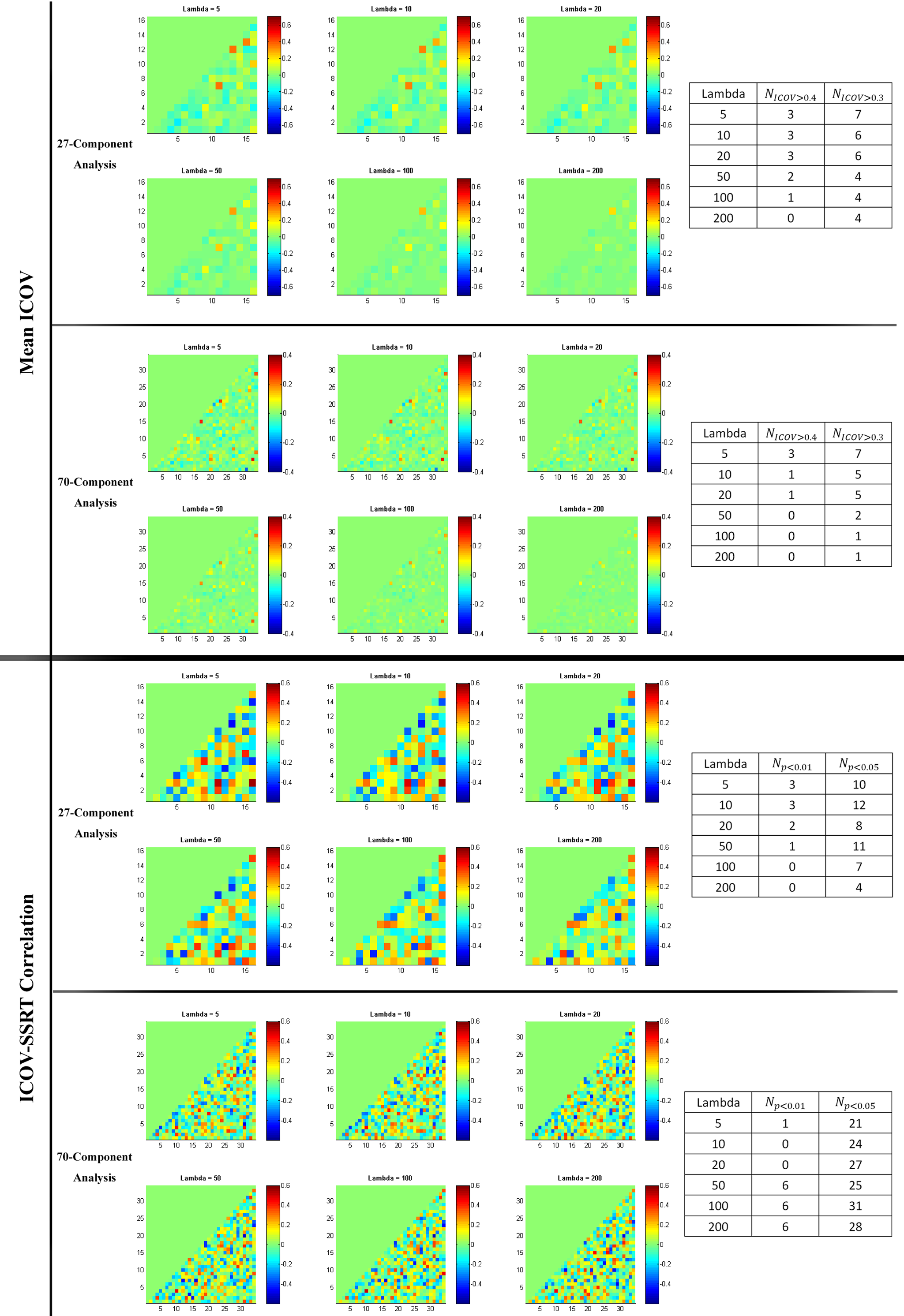

Supplement: Figure S1 — Maps of the mean (across subjects) ICOV matrices (Upper) and the ICOV-vs-SSRT correlations (Lower) acquired with different lambdas. The coordinates in each subfigure indicate the number of the component within the set of non-artefactual components. The correspondence between the component numbers and their coordinates in the subfigures can be found in Table S2. and indicate the number of mean-ICOVs larger than 0.4 and 0.3, respectively. and indicate the number of significant ICOV-vs-SSRT correlations at thresholds of p<0.01 (uncorrected) and p<0.05 (uncorrected), respectively. It can be seen that the mean ICOV matrices evaluated based on lambdas of 5, 10 and 20 are generally larger in magnitude and less sparse than those based on 50, 100, and 200. Based on 27-component analysis, more significant ICOV-vs-SSRT correlations were found with lambdas of 5, 10 and 20 (compared with 50, 100 and 200), and the strength of the correlations based on 70-component analysis were approximately the same between different lambdas. In this paper, results based on lambda = 10 were reported in detail. (TIF) [file pone.0066572.s001.tif]

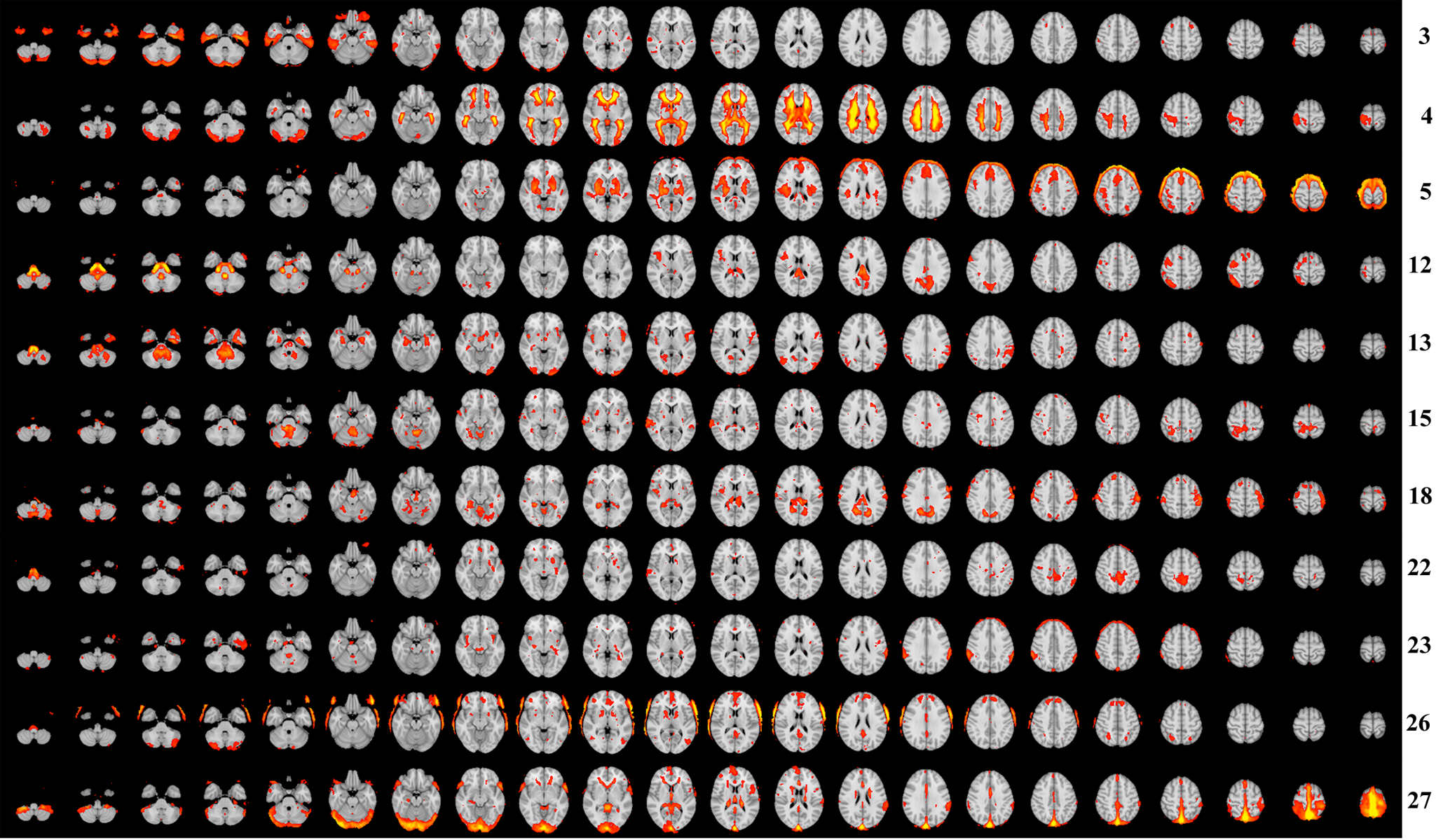

Supplement: Figure S2 — Maps of 11 components regarded to be associated with artifactual sources from the 27-component analysis. This figure shows every 3rd axial slice in 2-mm MNI152 standard space, starting with the lowest slice at −54 mm. On the right is the number of each component, which was based on the ranking of variance explained by the component. (TIF) [file pone.0066572.s002.tif]

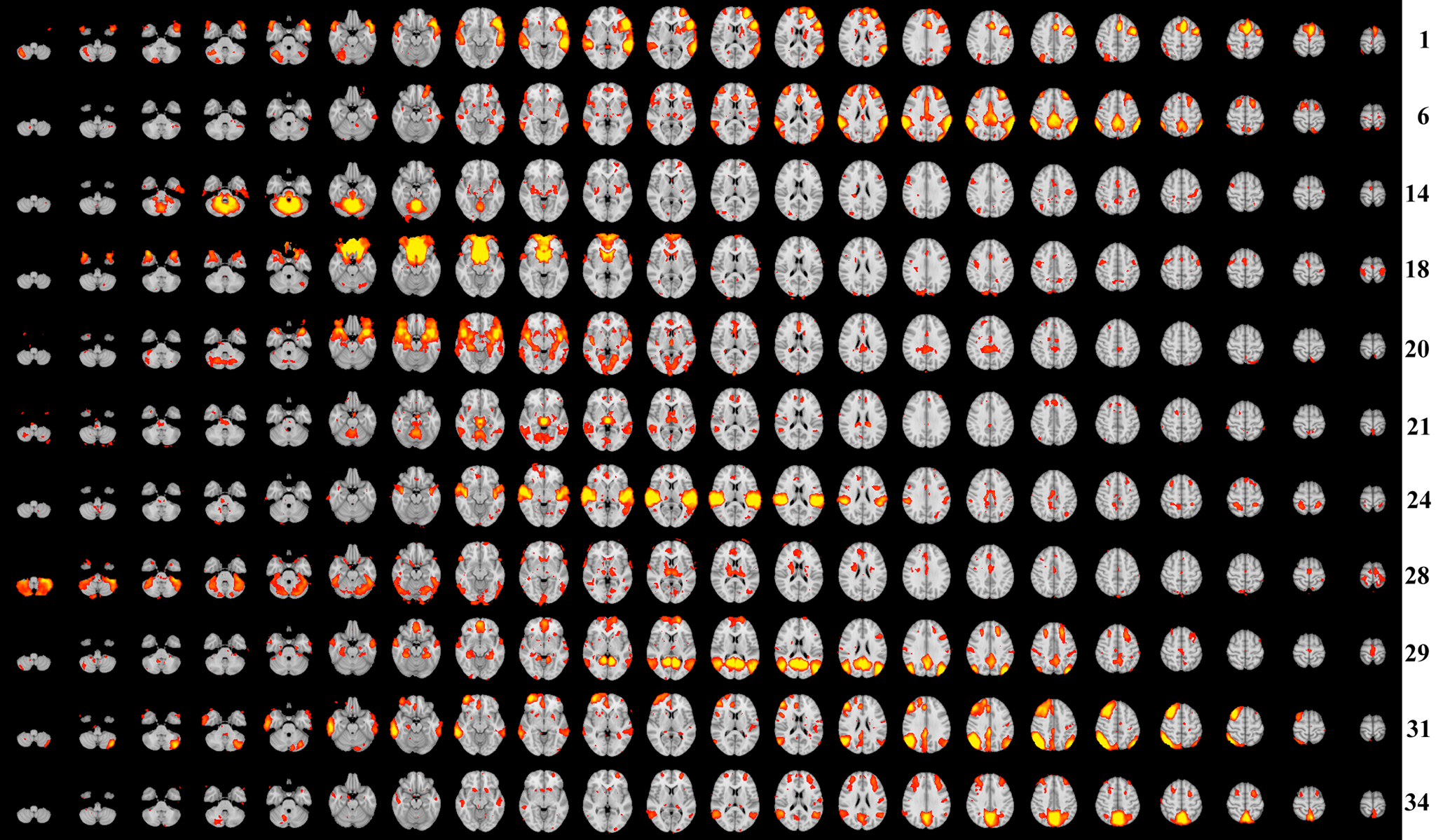

Supplement: Figure S3 — Maps of 11 of components from the 70-component analysis. These components were considered non-artefactual, but not shown in Fig. 1(B) in the main text. This figure shows every 3rd axial slice in 2-mm MNI152 standard space, starting with the lowest slice at −54 mm. On the right is the number of each component, which was based on the ranking of variance explained by the component. (TIF) [file pone.0066572.s003.tif]

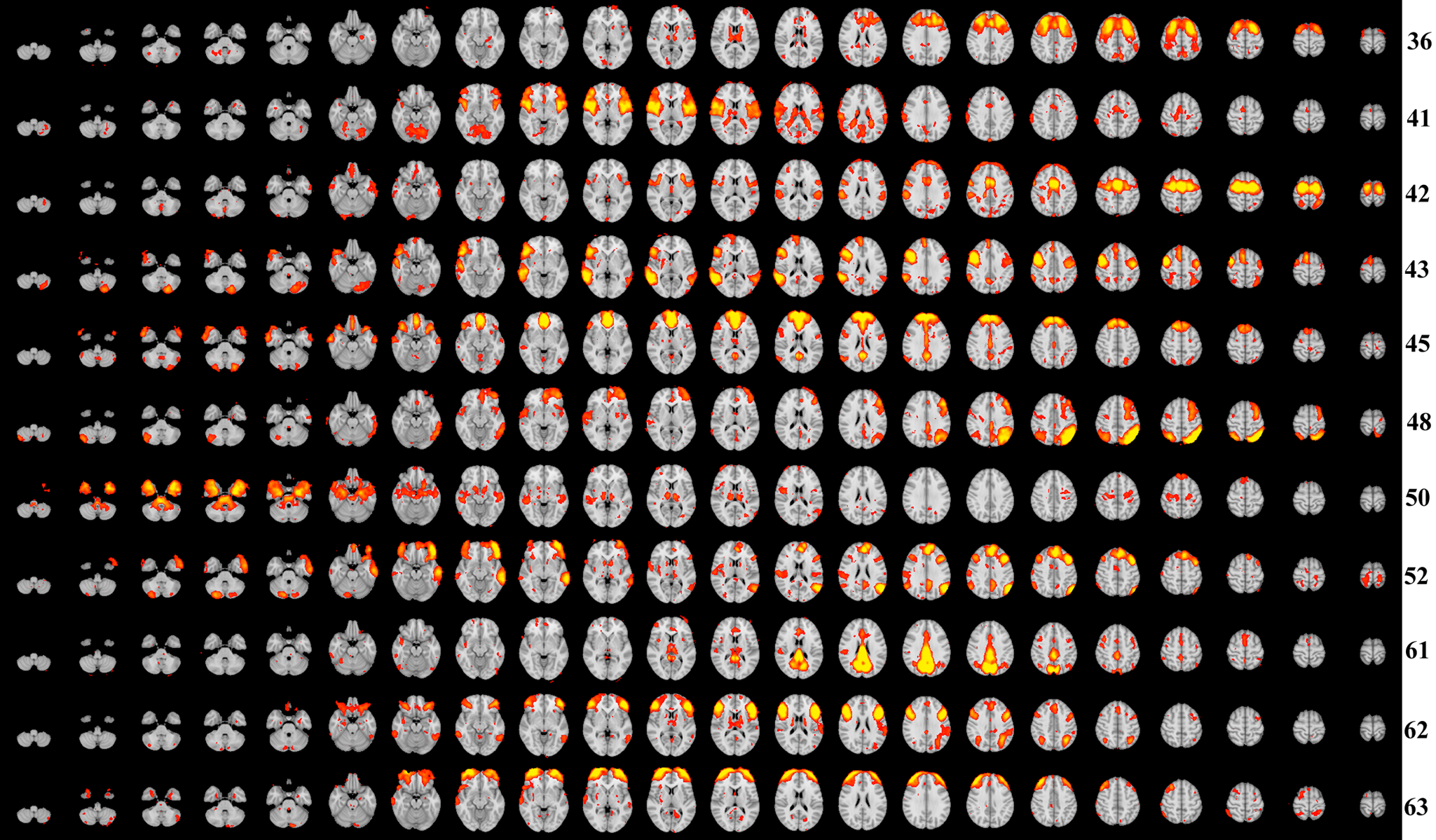

Supplement: Figure S4 — Maps of a further 11 components from the 70-component analysis. These components were considered non-artefactual, but not shown in Fig. 1(B) in main text. This figure shows every 3rd axial slice in 2-mm MNI152 standard space, starting with the lowest slice at −54 mm. On the right is the number of each component, which was based on the ranking of variance explained by the component. (TIF) [file pone.0066572.s004.tif]

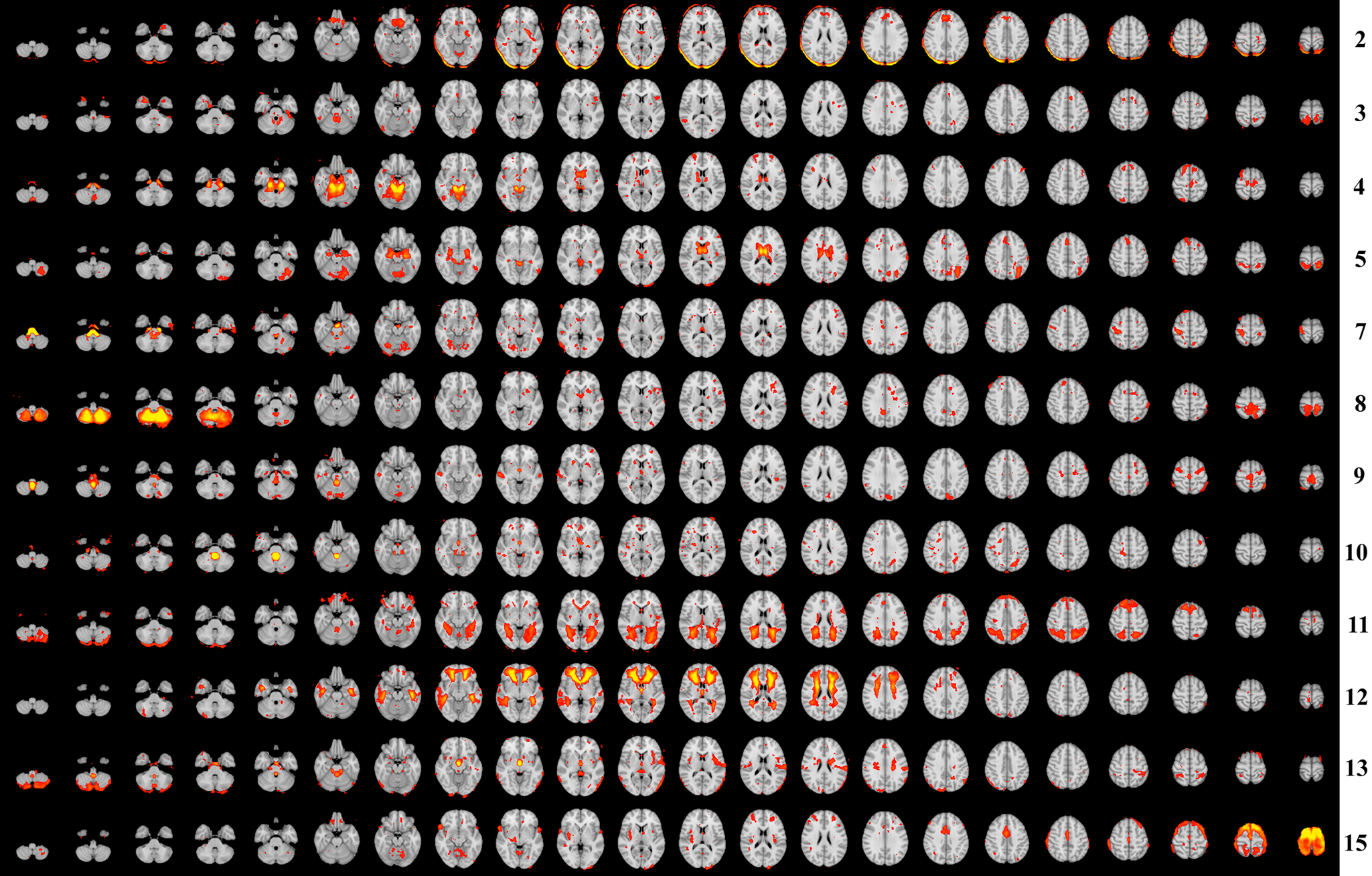

Supplement: Figure S5 — Maps of 12 of 36 components regarded to be associated with artifact sources from the 70-component analysis. This figure shows every 3rd axial slice in 2-mm MNI152 standard space, starting with the lowest slice at −54 mm. On the right is the number of each component, which was based on the ranking of variance explained by the component. (TIF) [file pone.0066572.s005.tif]

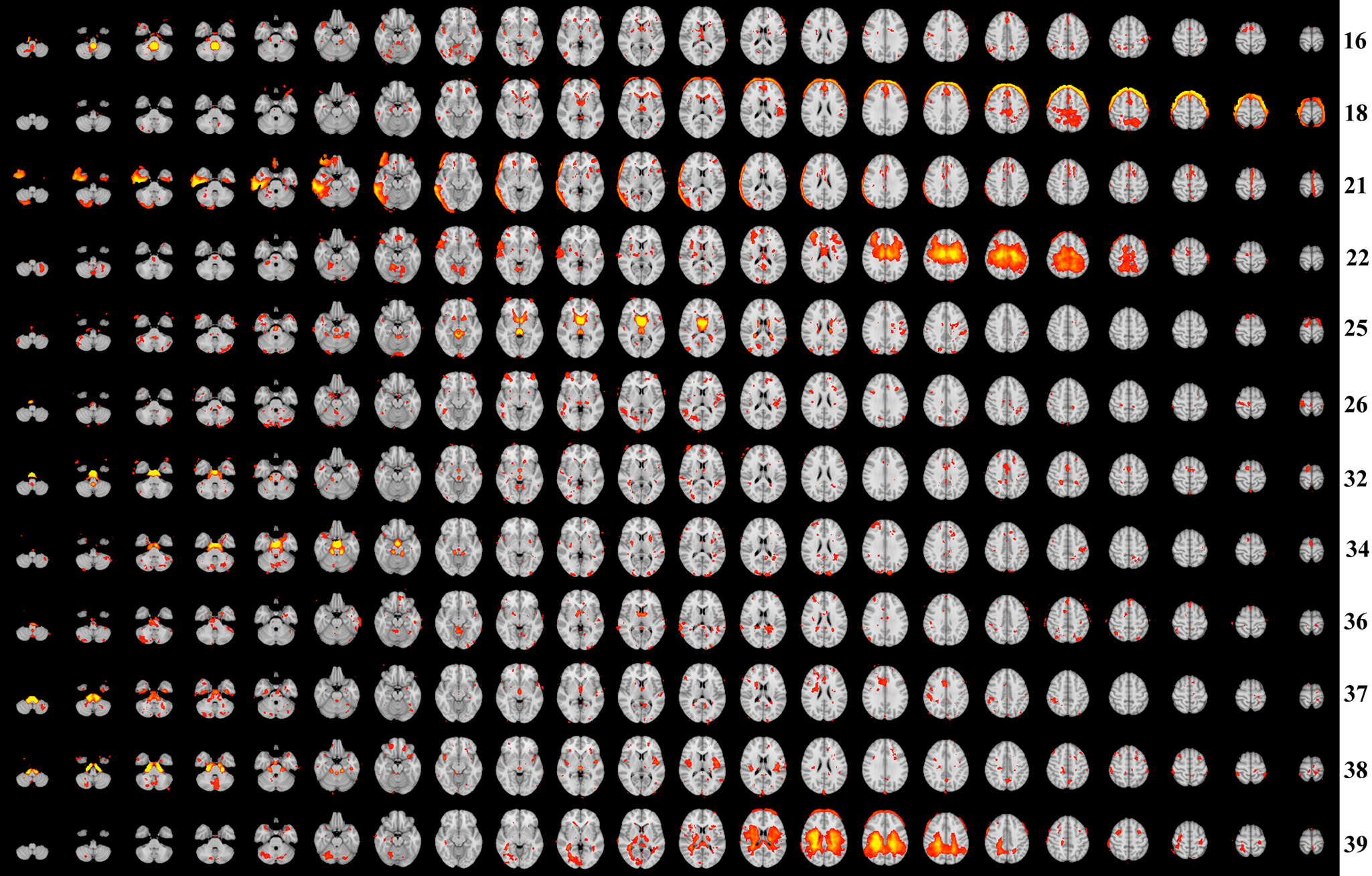

Supplement: Figure S6 — Maps of a further 12 of 36 components regarded to be associated with artifact sources from the 70-component analysis. This figure shows every 3rd axial slice in 2-mm MNI152 standard space, starting with the lowest slice at −54 mm. On the right is the number of each component, which was based on the ranking of variance explained by the component. (TIF) [file pone.0066572.s006.tif]

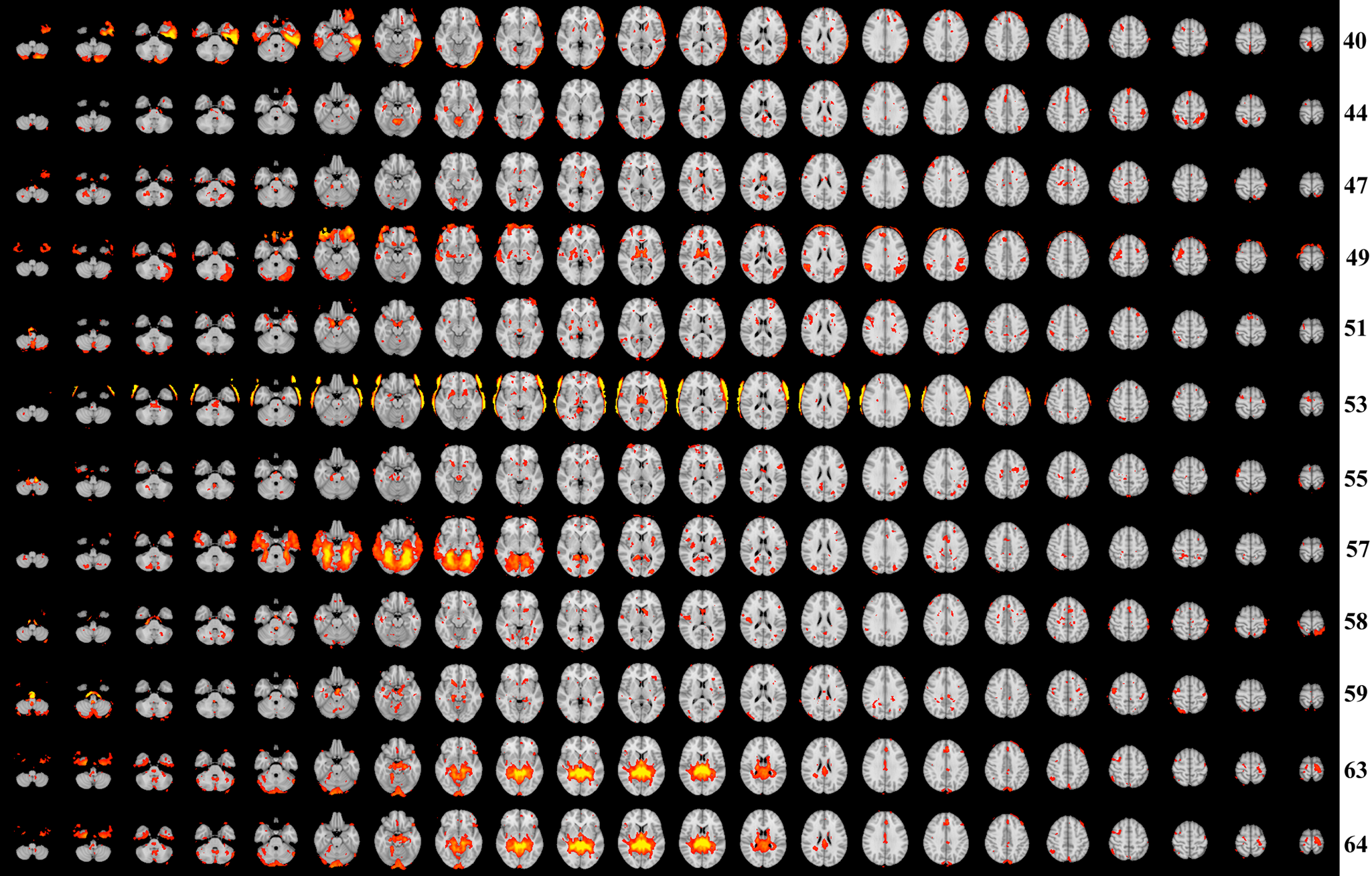

Supplement: Figure S7 — Maps of a further 12 of 36 components regarded to be associated with artifact sources from the 70-component analysis. This figure shows every 3rd axial slice in 2-mm MNI152 standard space, starting with the lowest slice at −54 mm. On the right is the number of each component, which was based on the ranking of variance explained by the component. (TIF) [file pone.0066572.s007.tif]

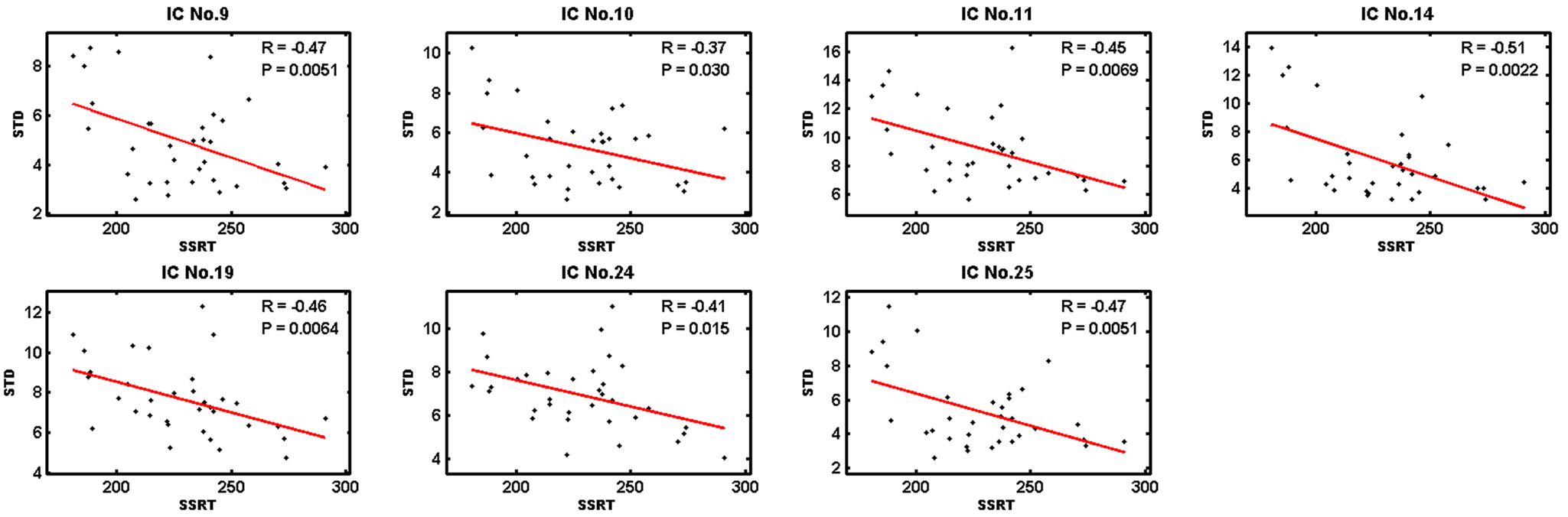

Supplement: Figure S8 — Plots of significant negative cross-subject correlation of RSN timeseries amplitude (standard deviation) vs. SSRT, based on 27-component analysis. The threshold was p<0.05 (FDR corrected), which corresponds to uncorrected . SSRT correlation of component No. 10, which did not survive thresholding, is also shown because its counterparts obtained at 70-dimensions exhibited significant SSRT correlation (see Table 1 in the main text for more details). (TIF) [file pone.0066572.s008.tif]

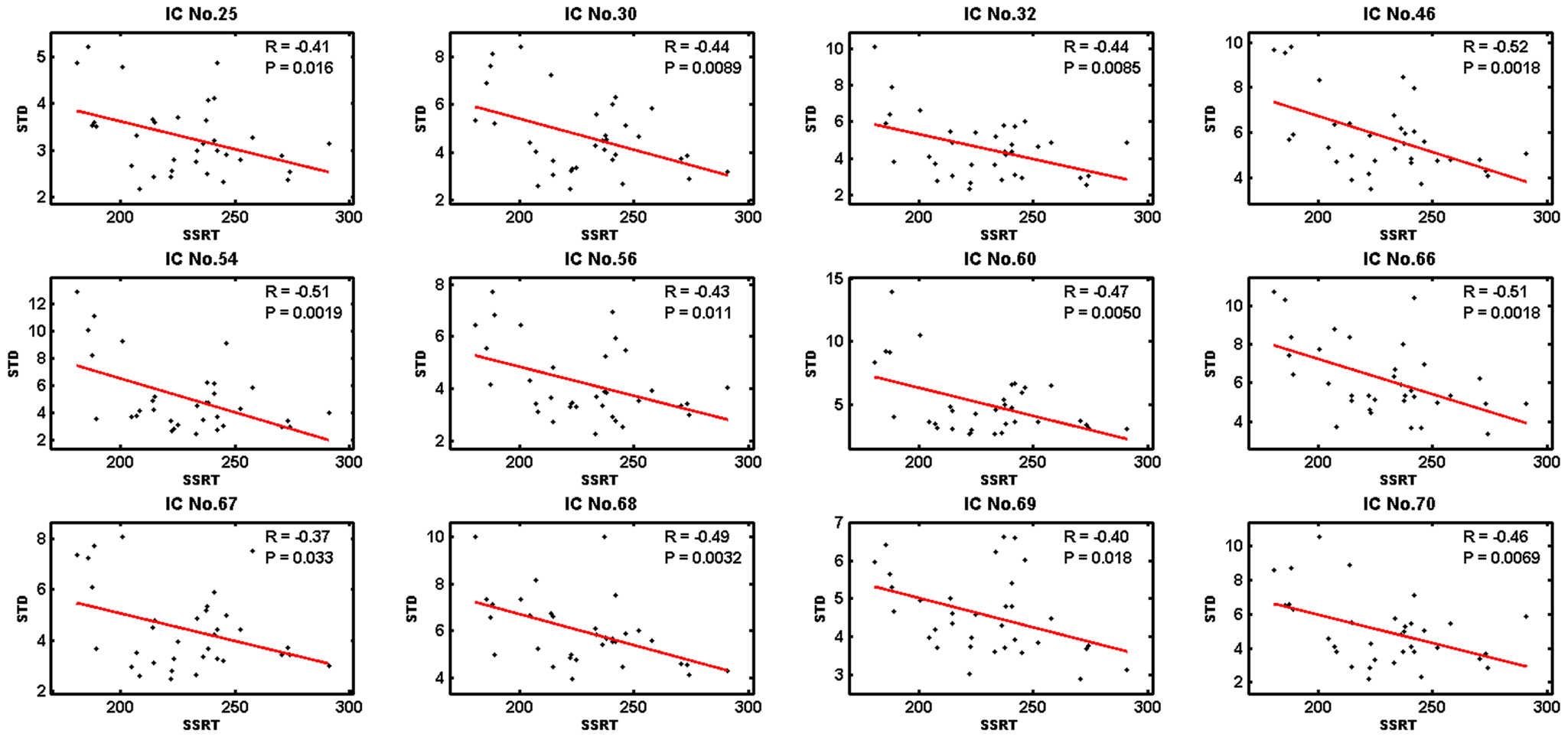

Supplement: Figure S9 — Plots of significant negative cross-subject correlation of RSN timeseries amplitude (standard deviation) vs. SSRT, based on 70-component analysis. The threshold was p<0.05 (FDR corrected), which corresponds to uncorrected . SSRT correlations of component Nos. 25, 67, 69, which did not survive thresholding, are also shown because their counterparts obtained at 27-dimension exhibited significant SSRT correlation (see Table 1 in the main text for more details). (TIF) [file pone.0066572.s009.tif]

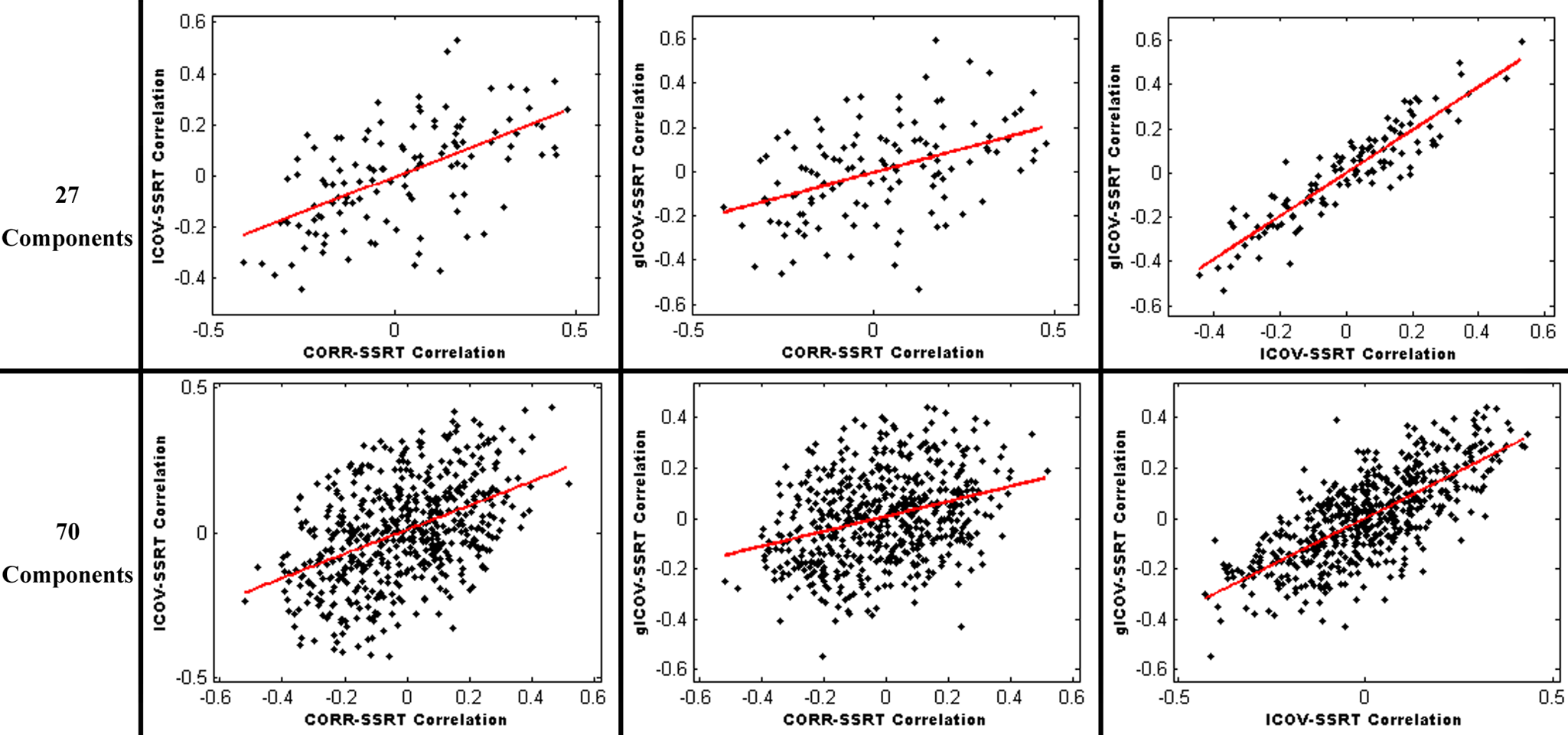

Supplement: Figure S10 — Similarities between different methods for estimating RSN timerseries correlation matrices. The correlations between SSRT and matrix elements is estimated for each method, and then compared between methods. It can be seen that the results based on the two partial-correlation methods (ICOV & gICOV) shared much resemblance to each other (the right column). (TIF) [file pone.0066572.s010.tif]

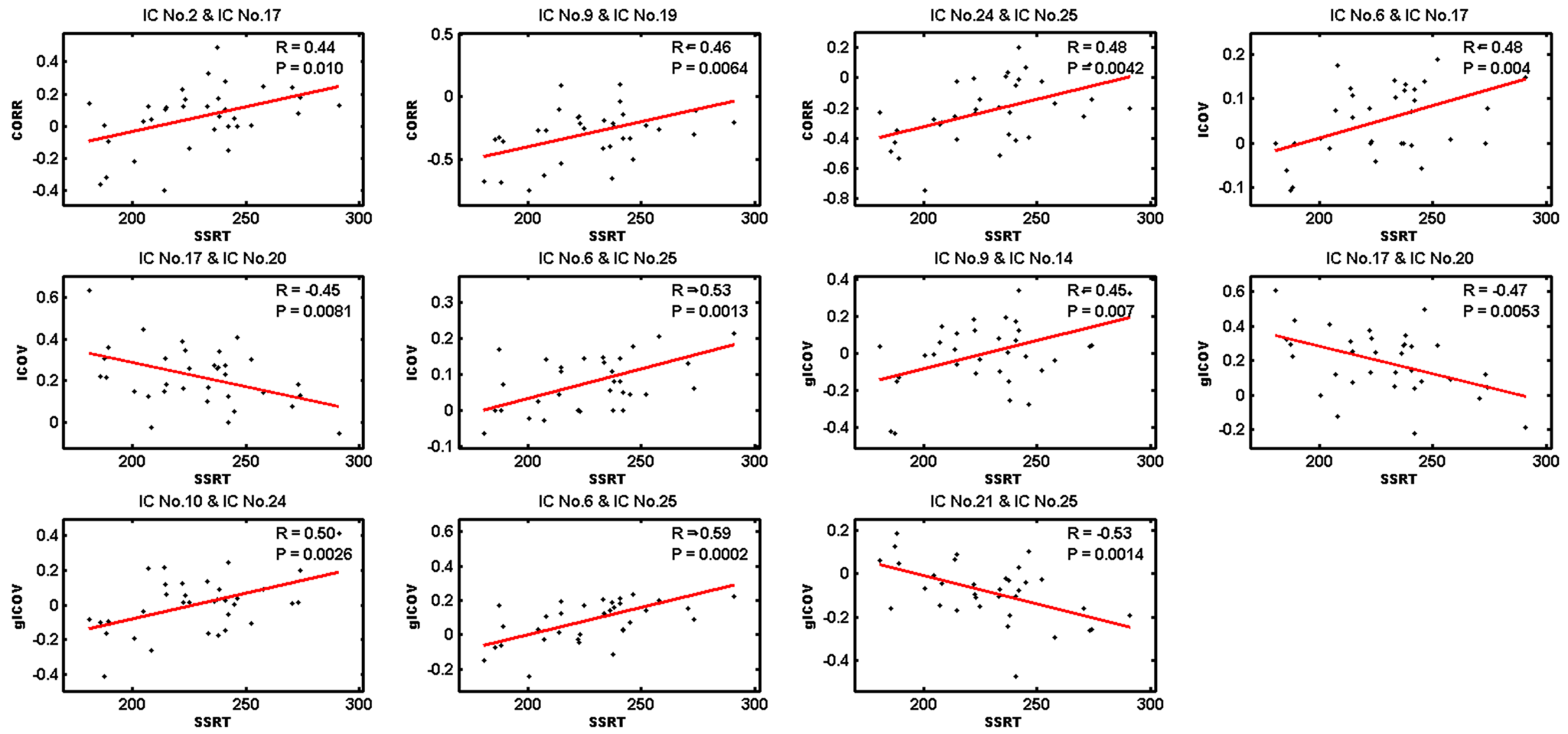

Supplement: Figure S11 — SSRT and network matrix correlations surviving an uncorrected threshold of p <0.10 based on the 27-component analysis. Only gICOV (6, 25) was fully significant (p<0.05, FDR corrected). (TIF) [file pone.0066572.s011.tif]

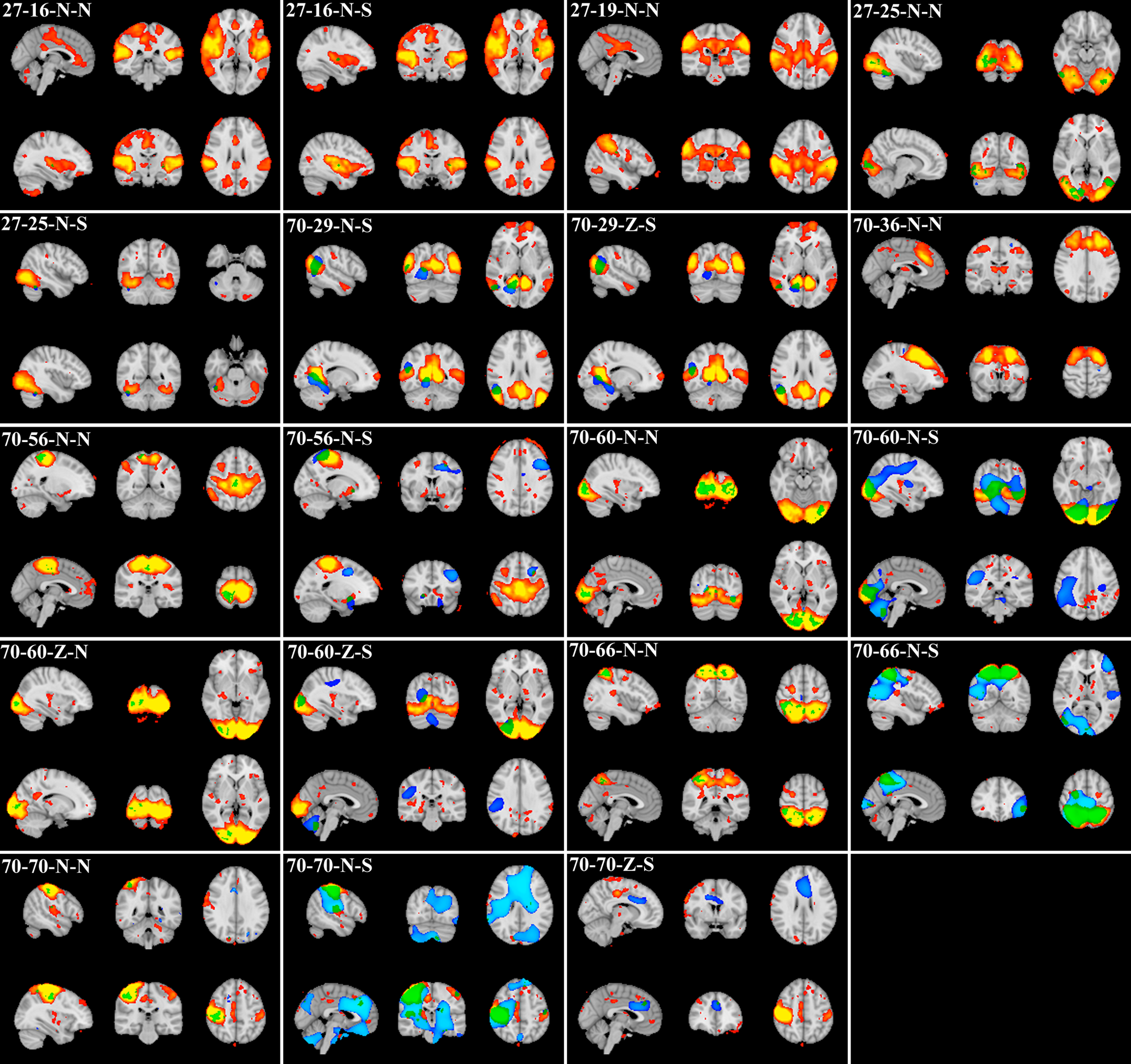

Supplement: Figure S12 — Maps of other significant negative spatial-map-vs-SSRT correlations besides those shown in Fig. 4 in the main text. The threshold was p<0.05 (TFCE enhanced, FWE corrected). The correlation maps were superimposed on their respective group-mean spatial maps obtained by Group-ICA and then on the MNI152 template. The group-mean spatial maps were provided here to give illusive idea about whether the significant regions lie within or outside the RSNs. Red-yellow indicates significant regions in the group-mean RSN map, blue indicates significant spatial-map-vs-SSRT correlations, and green color indicates overlap between the two. Each subfigure was identified by an array of “Number-Number-Character-Character”. The first number of this array indicates the dimension of ICA decomposition; the second number indicates the component number; the first character indicates whether the spatial maps were z-transformed before spatial-map-vs-SSRT correlation: with “N” indicates NO z-transformation and “Z” indicates the opposite; the second character indicates whether the spatial maps were 10-mm spatially smoothed before spatial-map-vs-SSRT correlation: with “N” indicating NO spatial smoothing and “S” indicating the opposite. For instance, “27-16-N-N” indicates that the subfigure was based on the unsmoothed non-z-transformed spatial map of the component No. 16 based on 27-component analysis. (TIF) [file pone.0066572.s012.tif]
