# Supplementary material for: Spatial vs. Temporal Features in ICA of Resting-State fMRI – A Quantitative and Qualitative Investigation in the Context of Response Inhibition
Source: PLoS One. 2013 Jun 18;8(6):e66572. doi: 10.1371/journal.pone.0066572 (PMC3688987; doi:10.1371/journal.pone.0066572)
Supplement: Table S1 — Summary of each network from the 27-component analysis, and those exhibiting significant timeseries amplitude-vs-SSRT correlations from the 70-component analysis. The IC No. was based on the ranking of variance explained by the component. (DOCX) [file pone.0066572.s013.docx]

| ***27-Component Analysis*** | | | |
| --- | --- | --- | --- |
| **IC No.** | **Network function** | **IC No.** | **Network function** |
| 1 | Executive control | 14 | Primary-medial visual |
| 2 | Action control | 16 | Auditory-motor |
| 6 | Orbital frontal | 17 | Spatial processing |
| 7 | Posterior default mode | 19 | Secondary dorsal attention |
| 8 | Somatosensory | 20 | Language |
| 9 | Motor control | 21 | Anterior default mode |
| 10 | Motor | 24 | Task-activation |
| 11 | Primary dorsal attention | 25 | Primary-occipital / higher visual |
| ***70-Component Analysis*** | | | |
| **IC No.** | **Network function** | **IC No.** | **Network function** |
| 25 | Motor control | 60 | Lateral primary visual |
| 30 | Left Motor | 66 | Secondary dorsal attention |
| 32 | Motor | 67 | Higher visual |
| 46 | Dorsal Visual Stream | 68 | Primary dorsal attention |
| 54 | Medial primary Visual | 69 | Salience Processing |
| 56 | Motor | 70 | Right Motor |
