# Supplementary material for: Spatial vs. Temporal Features in ICA of Resting-State fMRI – A Quantitative and Qualitative Investigation in the Context of Response Inhibition
Source: PLoS One. 2013 Jun 18;8(6):e66572. doi: 10.1371/journal.pone.0066572 (PMC3688987; doi:10.1371/journal.pone.0066572)
Supplement: Table S2 — Correspondence between the component numbers and their coordinates in the subfigures of Figs. 2 and 3 in the main text and Fig. S1. The Component No. is based on the ranking of variance explained by the component. The Coordinate indicates the number of the component within the set of non-artefactual components. This table bridges the Component Nos. used in other parts of the paper and the Coordinates in Figs. 2, 3 and S1. According to this table, for instance, the value at the coordinate (3, 14) in Fig. 2 based on 27 component-analysis indicates the mean correlation/partial-correlation between the components No. 6 and No. 21. (DOCX) [file pone.0066572.s014.docx]

| ***27-Component Analysis*** | | | | | | | | | | | | |
| --- | --- | --- | --- | --- | --- | --- | --- | --- | --- | --- | --- | --- |
| **Coordinate** | 1 | 2 | 3 | 4 | 5 | 6 | 7 | 8 | 9 | 10 | 11 | 12 |
| **Component No.** | 1 | 2 | 6 | 7 | 8 | 9 | 10 | 11 | 14 | 16 | 17 | 19 |
| **Coordinate** | 13 | 14 | 15 | 16 |  |  |  |  |  |  |  |  |
| **Component No.** | 20 | 21 | 24 | 25 |  |  |  |  |  |  |  |  |
| ***70-Component Analysis*** | | | | | | | | | | | | |
| **Coordinate** | 1 | 2 | 3 | 4 | 5 | 6 | 7 | 8 | 9 | 10 | 11 | 12 |
| **Component No.** | 1 | 6 | 14 | 18 | 20 | 21 | 24 | 25 | 28 | 29 | 30 | 31 |
| **Coordinate** | 13 | 14 | 15 | 16 | 17 | 18 | 19 | 20 | 21 | 22 | 23 | 24 |
| **Component No.** | 32 | 34 | 36 | 41 | 42 | 43 | 45 | 46 | 48 | 50 | 52 | 54 |
| **Coordinate** | 25 | 26 | 27 | 28 | 29 | 30 | 31 | 32 | 33 | 34 |  |  |
| **Component No.** | 56 | 60 | 61 | 62 | 63 | 66 | 67 | 68 | 69 | 70 |  |  |
