# Supplementary material for: Spatial vs. Temporal Features in ICA of Resting-State fMRI – A Quantitative and Qualitative Investigation in the Context of Response Inhibition
Source: PLoS One. 2013 Jun 18;8(6):e66572. doi: 10.1371/journal.pone.0066572 (PMC3688987; doi:10.1371/journal.pone.0066572)
Supplement: Text S1 — Description of Each Non-Artefactual Component from the 27-Component Analysis. (DOCX) [file pone.0066572.s015.docx]

## Description of Each Non-Artefactual Component from the 27-Component Analysis

Component No. 1 covers bilateral dorsal lateral prefrontal cortex, bilateral lateral orbital-frontal cortex, dorsal medial frontal cortex and bilateral temporal-parietal regions, and may correspond to an executive control network. Component No. 2 includes bilateral inferior frontal cortex, bilateral putamen, bilateral inferior parietal cortex and pre-SMA, and might be related to action control. Component No. 6, which covers the ventral medial prefrontal area, has formerly been observed in the study by Zuo et al. (2010). Component No. 7 covers the posterior cingulate cortex (PCC) / precuneus and the bilateral inferior parietal lobe, and corresponds to the posterior part of the default mode network (DMN). Component No. 8 is associated with somatosensory/motor processing, as identified by bilateral S1/M1 and thalamus. Component No. 9 includes regions critical for motor regulation and control, the SMA, bilateral putamen, thalamus and bilateral cerebellum, and corresponds to the motor control network. Component No. 10 covers the bilateral M1 and the supplementary motor area (SMA), and corresponds to the motor network. Components No. 11 and No. 19 are dorsal attention networks, with each including two critical components of the dorsal attention, bilateral intraparietal sulcus and the bilateral frontal eye field. Components No. 14 and No. 25 correspond to primary-medial and primary-occipital/higher visual networks, respectively. Component No. 16 includes the bilateral superior and medial temporal cortex, the right M1 and the SMA / dorsal anterior cingulated cortex (ACC), and corresponds to an auditory-motor network. Components No. 17 and No. 20 cover lateralized frontal-parietal areas, and correspond to possibly a visual-spatial processing network and the language network, respectively. Component No. 21 covers the ventral anterior ACC / medial frontal cortex, PCC and the bilateral temporal pole, and corresponds to the anterior part of the DMN. Component No. 24 covers bilateral frontal-parietal regions, the dorsal ACC and the orbital-frontoinsula regions, and corresponds to the task-activation network as proposed by Seeley et al. (2007).

**References**

*Seeley, W.W., Menon, V., Schatzberg, A.F., Keller, J., Glover, G.H., Kenna, H., Reiss, A.L., Greicius, M.D., 2007. Dissociable intrinsic connectivity networks for salience processing and executive control. J Neurosci 27, 2349-2356.*

[*Zuo, X.N*](http://www.ncbi.nlm.nih.gov/pubmed?term=%22Zuo%20XN%22%5BAuthor%5D)*.,* [*Kelly, C*](http://www.ncbi.nlm.nih.gov/pubmed?term=%22Kelly%20C%22%5BAuthor%5D)*.,* [*Adelstein, J.S*](http://www.ncbi.nlm.nih.gov/pubmed?term=%22Adelstein%20JS%22%5BAuthor%5D)*.,* [*Klein, D.F*](http://www.ncbi.nlm.nih.gov/pubmed?term=%22Klein%20DF%22%5BAuthor%5D)*.,* [*Castellanos, F.X*](http://www.ncbi.nlm.nih.gov/pubmed?term=%22Castellanos%20FX%22%5BAuthor%5D)*.,* [*Milham, M.P*](http://www.ncbi.nlm.nih.gov/pubmed?term=%22Milham%20MP%22%5BAuthor%5D)*., 2010. Reliable intrinsic connectivity networks: test-retest evaluation using ICA and dual regression approach.* [*Neuroimage*](http://www.ncbi.nlm.nih.gov/pubmed/19896537##) *49, 2163-2177.*
