# Supplementary material for: Spatial vs. Temporal Features in ICA of Resting-State fMRI – A Quantitative and Qualitative Investigation in the Context of Response Inhibition
Source: PLoS One. 2013 Jun 18;8(6):e66572. doi: 10.1371/journal.pone.0066572 (PMC3688987; doi:10.1371/journal.pone.0066572)
Supplement: Text S2 — A Discussion of the Functional Significance of Some Spatial-Map-vs-SSRT Correlations. (DOCX) [file pone.0066572.s016.docx]

## A Discussion of the Functional Significance of Some Spatial-Map-vs-SSRT Correlations

Some significant SSRT correlations found through investigation of the spatial maps might well be unreported if only the timeseries are investigated. For instance, significant spatial-map-vs-SSRT correlations were also detected in two regions of the DMN on the spatial map of the DMN, namely, the right inferior parietal lobue and the posterior cingulate cortex (see subfigures 70-29-N-S and 70-29-Z-S of Fig. S11). This result indicated that the strength of the interactions between DMN regions might also contribute to individual differences in response inhibition. This suggestion could be supported by former findings from the following two aspects: (1) the DMN has been reported to underlie a variety of functions such as stimulus-independent thought (McKiernan et al., 2006; Buckner et al., 2008), momentary lapses in attention (Weissman et al., 2006; Li et al., 2007) and spontaneous cognition (Buckner et al., 2008), and all of these processes tend to influence the individual cognitive task performance. (2) the strength of interaction between DMN regions has been reported to be able to predict individual cognitive task performance (Hampson et al., 2006). More closely related to the present analysis, Li et al. (2007) reported that the greater activity of the DMN regions before “Stop” signals could predict performance errors in the coming “Stop” trials.

Despite the interactions between networks and regions within themselves, the interaction between the dorsal anterior cingulate cortex (dACC) and the motor network (see subfigures 70-70-N-S and 70-70-Z-S of Fig. S11), as well as that between the visual regions and secondary DAN (see subfigures 70-66-N-S), were also observed to be related to individual differences in response inhibition. The dACC has repeatedly been reported to be active under situations which require conflict monitoring and error detection (for a review, see Botvinick et al., 2004). The present findings of significant spatial-map-vs-SSRT correlations in dACC on the spatial map of the motor network, as well as those in the visual regions on the spatial map of the secondary DAN indicate that the better cooperation between conflict monitoring and motor action execution, and cooperation between visual information processing and top-down control of attention resources (as carried out by the secondary DAN) would facilitate response inhibition.

**References**

*Botvinick, M.M., Cohen, J.D., Carter, C.S., 2004.* [*Conflict monitoring and anterior cingulate cortex: an update.*](http://www.ncbi.nlm.nih.gov/pubmed/15556023)*Trends Cogn Sci 8: 539-46.*

*Buckner, R.L., Andrews-Hanna, J.R., Schacter, D.L., 2008. The brain's default network: anatomy, function, and relevance to disease. Ann N Y Acad Sci 1124: 1–38.*

*Hampson, M., Driesen, N.R., Skudlarski, P., Gore, J.C., Constable, R.T., 2006. Brain connectivity related to working memory performance. J Neurosci 26, 13338-13343.*

*Li, C.S., Yan, P., Bergquist, K.L., Sinha, R., 2007. Greater activation of the "default" brain regions predicts stop signal errors. Neuroimage 38, 640-648.*

*McKiernan, K.A., D'Angelo, B.R., Kaufman, J.N., Binder, J.R., 2006. Interrupting the "stream of consciousness": an fMRI investigation. Neuroimage 29, 1185-1191.*

*Weissman, D.H., Roberts, K.C., Visscher, K.M., Woldorff, M.G., 2006. The neural bases of momentary lapses in attention. Nat Neurosci 9, 971-978.*
